# The Eukaryotic-Like Ser/Thr Kinase PrkC Regulates the Essential WalRK Two-Component System in *Bacillus subtilis*

**DOI:** 10.1371/journal.pgen.1005275

**Published:** 2015-06-23

**Authors:** Elizabeth A. Libby, Lindsie A. Goss, Jonathan Dworkin

**Affiliations:** Department of Microbiology & Immunology, College of Physicians and Surgeons, Columbia University, New York, New York, United States of America

## Abstract

Most bacteria contain both eukaryotic-like Ser/Thr kinases (eSTKs) and eukaryotic-like Ser/Thr phosphatases (eSTPs). Their role in bacterial physiology is not currently well understood in large part because the conditions where the eSTKs are active are generally not known. However, all sequenced Gram-positive bacteria have a highly conserved eSTK with extracellular PASTA repeats that bind cell wall derived muropeptides. Here, we report that in the Gram-positive bacterium *Bacillus subtilis*, the PASTA-containing eSTK PrkC and its cognate eSTP PrpC converge with the essential WalRK two-component system to regulate WalR regulon genes involved in cell wall metabolism. By continuously monitoring gene expression throughout growth, we consistently find a large PrkC-dependent effect on expression of several different WalR regulon genes in early stationary phase, including both those that are activated by WalR (*yocH*) as well as those that are repressed (*iseA*, *pdaC*). We demonstrate that PrkC phosphorylates WalR *in vitro* and *in vivo* on a single Thr residue located in the receiver domain. Although the phosphorylated region of the receiver domain is highly conserved among several *B. subtilis* response regulators, PrkC displays specificity for WalR *in vitro*. Consistently, strains expressing a nonphosphorylatable WalR point mutant strongly reduce both PrkC dependent activation and repression of *yocH*, *iseA*, and *pdaC*. This suggests a model where the eSTK PrkC regulates the essential WalRK two-component signaling system by direct phosphorylation of WalR Thr101, resulting in the regulation of WalR regulon genes involved in cell wall metabolism in stationary phase. As both the eSTK PrkC and the essential WalRK two-component system are highly conserved in Gram-positive bacteria, these results may be applicable to further understanding the role of eSTKs in Gram-positive physiology and cell wall metabolism.

## Author Summary

A central question in bacterial physiology is how bacteria sense and respond to their environment. The archetype of bacterial signaling systems is the two-component signaling system composed of a sensor protein histidine kinase that activates a transcription factor response regulator in response to a specific signal. In addition, bacteria also have signaling systems composed of eukaryotic-like Ser/Thr kinases and phosphatases. Even though these systems do not have dedicated transcription factors, they are capable of affecting gene expression. Here we show that a eukaryotic-like Ser/Thr kinase conserved in all sequenced Gram-positive bacteria converges with an essential two-component signaling system to regulate gene expression in the model organism *Bacillus subtilis*. We show that this eukaryotic-like Ser/Thr kinase phosphorylates the response regulator of a highly conserved and essential two-component signaling system, thereby increasing its activity. This phosphorylation results in the regulation of genes involved in the essential process of cell wall metabolism. Given that bacterial cell wall metabolism is the target of many known antibiotics, and mutations in both of these signaling systems change the antibiotic sensitivity of a number of important Gram-positive pathogens, we expect that our analysis will suggest novel insight into the emergence of antibiotic resistance.

## Introduction

Regulatory Ser/Thr phosphorylation has been assumed to be largely absent from prokaryotes. However, recently, phosphoproteomic analysis has identified numerous (~50) proteins phosphorylated on Ser or Thr residues in both Gram-positive and Gram-negative bacteria [1]. Likely candidates for the enzymes responsible for these modifications are bacterial proteins with structural and mechanistic similarities to eukaryotic Ser/Thr kinases (eSTKs) and phosphatases (eSTPs) [2,3]. These enzymes have been identified in phylogenetically diverse bacteria, and eSTKs may be directly responsible for a number of the observed Ser/Thr phosphorylations. Thus, how these kinases regulate cellular physiology remains unclear, but their conservation across the majority of sequenced bacterial species suggests that they play an important role.

Regulatory phosphorylation in bacteria has largely been studied in the context of two-component systems (TCS) that govern the expression of a regulon in response to a physiological signal. These signaling systems include a sensor histidine kinase, which when activated transphosphorylates a cognate response regulator, typically a DNA-binding protein, on a conserved aspartate residue. Once phosphorylated, the response regulator dimerizes and binds specific sequences in the promoters of the genes in its regulon. Hints that eSTKs might similarly be involved in transcriptional regulation come from studies demonstrating that mutations in eSTKs cause large-scale changes in gene expression [4–6]. However, in contrast to TCS systems which typically use response regulator phosphorylation to regulate gene expression, eSTKs and the eSTPs generally lack identified DNA binding motifs, suggesting they likely mediate changes in gene expression indirectly. Although eSTKs and eSTPs are also not generally associated with a specific partner transcription factor, at least some of the observed transcriptional changes may be due to direct phosphorylation of a transcription factor. As the regulons governed by some TCSs have been extensively studied, overlap between transcriptional changes caused by mutations in eSTKs and eSTPs and certain TCS systems has been observed [7,8]. A plausible mechanism for these effects is that an eSTK phosphorylates components of a TCS system and thereby affecting gene expression. Consistently, eSTKs from a number of organisms have been shown to phosphorylate TCS proteins, and these modifications affect expression of genes in the respective TCS regulon [7,8].

All sequenced Gram-positive bacteria contain a single membrane-bound eSTK comprised of an extracellular domain composed of three or four PASTA repeats and a cytoplasmic domain with extensive homology to eukaryotic Ser/Thr kinases [3,9]. The PASTA (**P**eptidoglycan binding **a**nd **S**er/**T**hr kinase **a**ssociated) motif was originally identified in penicillin-binding proteins that are responsible for peptidoglycan polymerization, suggesting that it mediates peptidoglycan recognition [10]. Consistent with this hypothesis, isolated PASTA domains interact *in vitro* with a peptide corresponding to the peptidoglycan stem peptide [11–13]. In the Gram-positive bacterium *B*. *subtilis*, the PASTA-containing eSTK is PrkC, which is required for spore germination [14] and for regulation of the production of a secreted muramidase [15] in response to muropeptides. PrkC is transcribed during all phases of growth, and is co-transcribed with the eSTP PrpC [16]. Thus, PrkC could play a role in the regulation of peptidoglycan metabolism in *B*. *subtilis*.

A signaling system that is known to be important for peptidoglycan metabolism is the WalRK TCS, a well conserved, essential TCS found in low-GC-Gram-positive bacteria including *B*. *subtilis* [17–19]. Mutations in the response regulator WalR that alter its function significantly result in the generation of *B*. *subtilis* L-forms, variants that lack cell wall [20]. The essentiality of WalR likely derives from its regulation of at least one WalRK regulon member, although the identity of that gene, or combination of genes, is not known for most WalRK-containing species (see *S*. *pneumoniae* for an exception [21]). This essentiality is unusual; WalRK is the only essential TCS (out of 34) in *B*. *subtilis* [17]. The core WalRK regulon conserved in different species comprises ~10 genes involved in cell wall metabolism, including several peptidoglycan hydrolases [18]. In *B*. *subtilis*, ~20 WalRK regulon genes have been identified through several approaches including the use of a hybrid regulator [22], an IPTG-inducible WalRK operon [23,24], and ChIP [25]. Differential regulation of WalRK regulated genes is observed upon treatment with various cell wall-targeting antibiotics, suggesting that the WalK activating ligand is a molecule derived from the cell wall [18].

As the eSTK PrkC and the TCS WalRK have functional overlap, it is plausible that these two types of signaling systems converge in Gram-positive bacteria. Consistent with this hypothesis, deletion of the PrkC homologs in *S*. *pneumoniae* (StkP; [5]), *S*. *mutans* (PknB; [6]), *S*. *pyogenes* (Stk; [26]) and *S*. *aureus* (PknB; [4]) resulted in the down-regulation of genes known to be under control of either WalR or its homolog VicR. In the model Gram-positive *B*. *subtilis*, *yocH*, a well-characterized member of the WalRK regulon encoding a cell wall hydrolase, is regulated by both WalR and PrkC. WalR directly binds the *yocH* promoter *in vitro* [22], and *yocH* transcription is activated by WalR expression *in vivo* [27]. Intriguingly, *yocH* also demonstrates PrkC-dependent activation in response to cell wall-derived muropeptides [15]. Thus, these data suggest a functional interaction between PrkC and the WalRK TCS. Here we use *in vitro* and *in vivo* approaches to demonstrate that, consistent with this hypothesis, PrkC regulates the WalRK TCS through phosphorylation of WalR, resulting in the regulation of genes involved in cell wall metabolism by an eSTK.

## Results

### PrkC regulates *yocH* transcription in stationary phase

**Fig 1:**
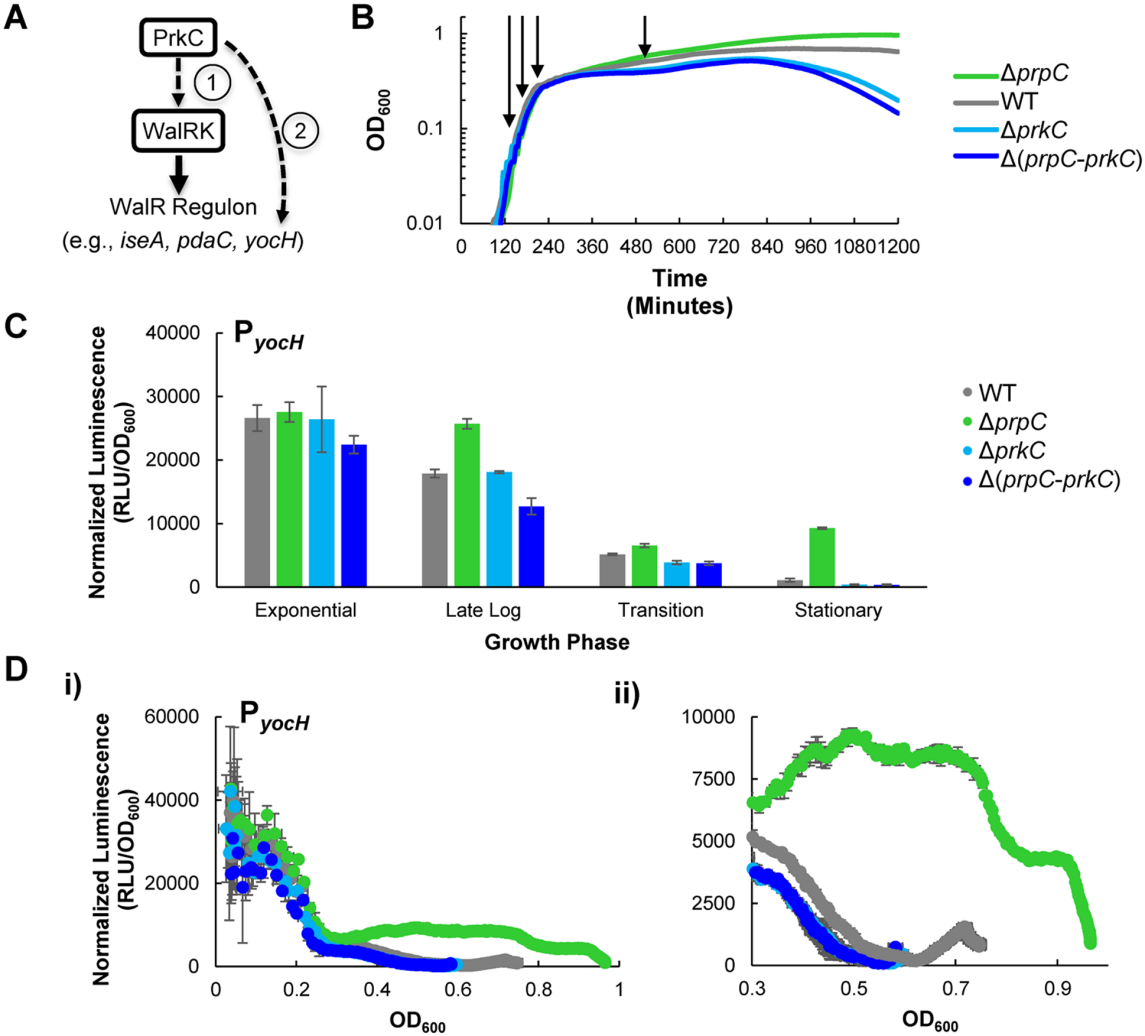
The kinase PrkC and the phosphatase PrpC regulate the WalRK regulon gene *yocH*. **A)** Models of PrkC activation of the WalRK regulon gene *yocH*. (1) PrkC acts via the WalRK system on *yocH*, thereby also affecting other WalRK regulon genes. (2) Alternately, PrkC acts through another mechanism on *yocH*. **B)** Growth curves of WT, Δ*prpC*, Δ*prkC*, and Δ(*prpC-prkC*) strains in LB. 150 μl cultures were grown in 96-well plates and OD_600_ was measured every 5 minutes. Under these conditions, strains lacking the kinase (‘Δ*prkC’*), or both the kinase and phosphatase (‘Δ(*prpC-prkC*)’), exhibit reduced survival in late stationary phase. Strains lacking only the phosphatase (‘Δ*prpC’*) have increased survival. No difference between any of the strains is observed in log phase. Shown are typical growth curves, plotted as mean OD_600_ as a function of time post-inoculation. Two shades of blue are used to group by color both strains lacking the kinase to highlight their similarity. **C)** Activation of *yocH* by PrkC in stationary phase. Transcriptional changes in *yocH* expression during exponential, late log, transition, and stationary phases in WT, Δ*prpC*, Δ*prkC*, and Δ(*prpC-prkC*) backgrounds. Relative luminescence (RLU) of a P_*yocH*_-*lux* transcriptional reporter was measured at each growth phase indicated in panel **(B)** and normalized by OD_600_ to give normalized luminescence. In stationary phase, *yocH* is >20 fold activated in a Δ*prpC* background (green) relative to Δ(*prpC-prkC*) (dark blue). A ~3.5 fold difference is observed between WT (gray) and Δ(*prpC-prkC*) (dark blue) strains (shown in detail in S1 Fig). Growth phases picked for comparison of gene expression correspond to OD_600_~0.1 (‘Exponential’), 0.2 (‘Late Log’), 0.3 (‘Transition’), and 0.5 (‘Stationary’) phase prior to the onset of lysis) as indicated by arrows in (**B**). **D**) PrkC-dependent *yocH* activation begins at the exit from exponential phase. **i)** Normalized luminescence of P_*yocH*_-*lux* plotted as a function of OD_600_ values from exponential phase through stationary phase in WT, Δ*prpC*, Δ*prkC*, and Δ(*prpC-prkC*) backgrounds. Points are plotted to the maximum OD_600_ observed for each strain. **ii)** Detail of plot in **i)**, from OD_600_~0.3 through the max OD_600_ reached for each strain.

Prior work demonstrated that expression of the WalR regulon gene *yocH* is controlled both by WalR binding to P_*yocH*_ [22,27] and by PrkC through an unknown mechanism [15]. These observations suggest two possible mechanisms of PrkC dependent *yocH* activation: 1) PrkC acts on the WalRK TCS to regulate *yocH* and consequently other members of the WalR regulon, or 2) PrkC acts through another (unknown) mechanism on *yocH* (Fig 1A). The first hypothesis predicts that changes in PrkC activity resulting in the activation of *yocH* would also result in transcriptional changes across the WalR regulon, namely increased activation of genes activated by WalR, as well as increased repression of genes repressed by WalR. The second hypothesis predicts that changes in PrkC activity that activate *yocH* may have little, or no consistent, effect on the expression of other members of the WalR regulon.

To test these alternate hypotheses, we first determined when PrkC activity has the strongest effect on *yocH* expression. We constructed a transcriptional fusion of the *yocH* promoter region to the *luxABCDE* operon, originally from *P*. *luminescens*, optimized for expression in Gram-positive bacteria [28] and integrated it into an ectopic locus in the chromosome. As the luminescence signal from the *luxABCDE* operon is relatively unstable, it can be used to measure both increases and decreases in gene expression [29]. Therefore, we used the P_*yocH*_-*luxABCDE* transcriptional reporter (hereafter referred to as P_*yocH*_ *-lux*) to continuously monitor changes in *yocH* expression throughout growth in rich media (LB) in a plate reader. Under these conditions, the doubling time for the WT strain harboring the P_*yocH*_ *-lux* reporter is roughly 22 minutes in exponential phase, comparable to the optimal growth rate of *B*. *subtilis* in LB at 37°C. We measured *yocH* expression as well as the corresponding growth curves (OD_600_) in the plate reader every 5 minutes in four genetic backgrounds: wild type (‘WT’), Δ*prpC* (no phosphatase), Δ*prkC* (no kinase), and Δ(*prpC-prkC)* (neither phosphatase nor kinase). Under these conditions, the growth of these four strains is consistent with the previously reported stationary phase survival defect of Δ*prkC* strains and increased longevity of Δ*prpC* strains (Fig 1B) [30]. To highlight the consistent similarity between the Δ*prkC* and Δ(*prpC-prkC*) strains, indicating that the observed Δ*prpC* phenotypes are epistatic to *prkC*, Δ*prkC* mutants are grouped by color (Fig 1, shades of blue). Furthermore, these experiments also demonstrated that while there is no growth phenotype for Δ*prkC* and Δ*prpC* mutant strains during log phase growth, mild differences begin to appear during the transition to stationary phase and become more pronounced later in stationary phase prior to the onset of lysis (Fig 1B). Since transcriptional changes near the onset of lysis may be complicated to interpret, we chose to examine PrkC-dependent changes in gene expression at four characteristic growth points, as determined by position on the growth curve and corresponding measured OD_600_ (arrows indicate points for WT, Fig 1B): 1) exponential phase (OD_600_~0.1), 2) late log (OD_600_~0.2), 3) transition phase (OD_600_~0.3), and 4) stationary phase (OD_600_~0.5). We picked OD_600_~0.5 as a representative point for stationary phase measurements, as we observe comparable growth rates for all genetic backgrounds near this OD_600_, and it is prior to the onset of lysis. Note that OD_600_ values measured by the plate reader differ from those measured by a standard laboratory spectrophotometer. See materials and methods for more details.

To characterize PrkC-dependent *yocH* expression at each of these characteristic growth phases, we compared the normalized luminescence (relative luminescence, RLU, normalized by OD_600_) of the P_*yocH*_ *-lux* reporter in each genetic background at each characteristic growth phase (Fig 1C). Interestingly, although *prkC* and *prpC* are expressed throughout all growth phases [16], mutants in *prkC* and *prpC* did not exhibit strong changes in *yocH* expression until stationary phase (Fig 1C). To determine more precisely when in the growth cycle changes in *yocH* expression begin to appear, we plotted normalized luminescence as a function of OD_600_ for data acquired at 5 minute intervals for each genetic background (Fig 1D), yielding a synchronized picture of gene expression as a function of growth state. For clarity, these graphs show only the pre-lysis data (to maximum OD_600_) for each strain. In WT cells, *yocH* expression is high in exponential phase and falls in stationary phase (Fig 1C and 1D, gray), consistent with prior work [31]. Interestingly, this trend was reversed in a Δ*prpC* background beginning at the exit from log phase (OD_600_~0.3, Fig 1D, ii). This effect is PrkC dependent as both Δ*prkC* and Δ(*prpC-prkC*) strains show very similar expression profiles (compare shades of blue, Fig 1D). The WT expression profile (gray) lies between Δ*prpC* and Δ*prkC*, and is strongly biased towards Δ*prkC*. This suggests that under these conditions, PrkC exerts a relatively small average effect on *yocH* expression in WT cells, ~3.5 fold (S1 Fig), when compared to the total possible PrkC-dependent effect observed in a Δ*prpC* background. The difference in expression between Δ*prpC* and Δ*prkC* backgrounds suggests that PrkC is capable of exerting a >20 fold change on *yocH* expression in stationary phase.

### PrkC regulates the WalR regulon genes *iseA* and *pdaC*

**Fig 2:**
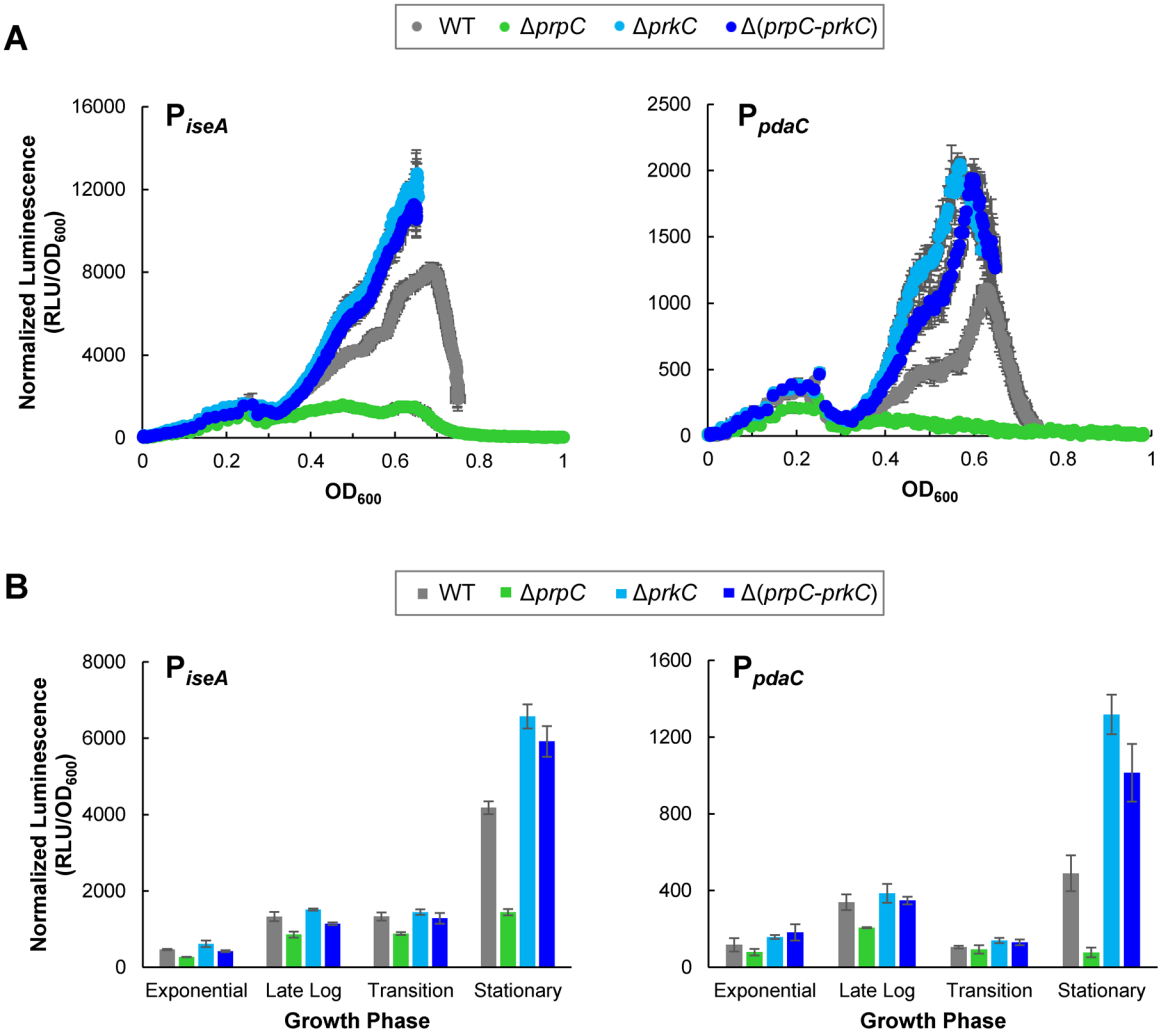
The PrkC kinase and the PrpC phosphatase regulate the WalR regulon genes *iseA* and *pdaC*. **A)** PrkC-dependent repression of *iseA* and *pdaC* is observed starting at the exit from exponential phase through stationary phase. Normalized luminescence as a function of OD_600_ throughout growth from exponential phase through stationary phase for P_*iseA*_-*lux* (left) and P_*pdaC*_ *-lux* (right). Points are plotted to the maximum OD_600_ observed for each strain. **B)** Comparison of PrkC-dependent changes in *iseA* (left) and *pdaC* (right) expression during exponential, late log, transition, and stationary phases. In stationary phase, *iseA* is ~4x repressed in a Δ*prpC* (green) background compared to a Δ(*prpC-prkC*) background (dark blue), and *pdaC* is ~13x repressed.

If PrkC regulates *yocH* expression through a WalR-dependent mechanism (Fig 1A), we would expect it to control expression of other genes in the WalR regulon. Two genes repressed by WalR were found to have the highest average WalR occupancy of their promoters in a ChIP study [25]: *iseA* (*yoeB*), encoding an inhibitor of autolysin activity [32], and *pdaC* (*yjeA*), a peptidoglycan deacetylase [33]. To determine if PrkC regulates *iseA* and *pdaC* under the same conditions where we observed PrkC-dependent regulation of *yocH*, we constructed P_*iseA*_-*luxABCDE* and P_*pdaC*_-*luxABCDE* transcriptional reporters. Using these reporters and the same growth conditions and techniques used to monitor *yocH* expression, we measured *iseA* and *pdaC* expression using normalized luminescence as a function of OD_600_ (Fig 2A). Strikingly, we observed that both *iseA* and *pdaC* exhibit strong PrkC-dependent repression at the same OD_600_ and growth phases where *yocH* exhibited PrkC-dependent activation. That is, beginning at the exit from log phase (OD_600_~0.3) and becoming most significant in stationary phase, both *iseA* and *pdaC* show increased repression in a Δ*prpC* background that is relieved in both Δ*prkC* and Δ(*prpC-prkC*) backgrounds (Fig 2A). We examined this growth phase dependence by extracting the normalized luminescence at four specific optical densities from the continuous data (Fig 2B). Similarly to what we observed for *yocH*, the WT strains have expression profiles in stationary phase between the Δ*prpC* and Δ*prkC* backgrounds, consistent with opposing regulation. In strains carrying a Δ*prpC* mutation, P_*iseA*_ *-lux* in stationary phase is expressed ~4x less and P_*pdaC*_ *-lux* is expressed >10x less than in strains carrying Δ(*prpC-prkC*) mutations (Fig 2B). As *yocH* is activated by WalR and both *iseA* and *pdaC* are repressed by WalR, these data strongly support a model (Fig 1A) where PrkC acts through the WalRK system to transcriptionally regulate *yocH*, *iseA*, *pdaC*, and other members of the WalR regulon.

### PrkC expression complements Δ*prkC* phenotypes

**Fig 3:**
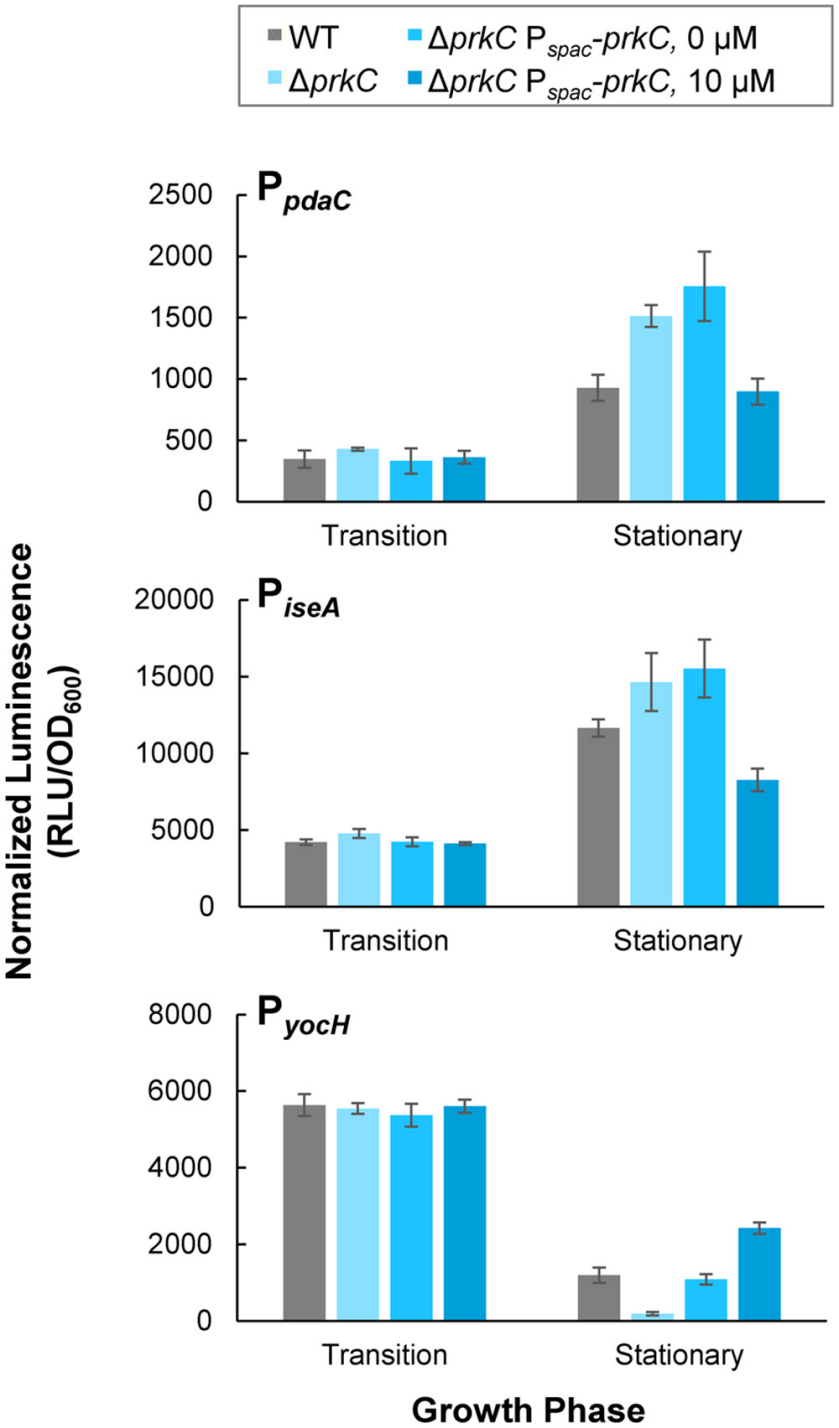
Inducible PrkC expression complements Δ*prkC* effects on expression of *WalR* regulon genes during stationary phase. Normalized luminescence was measured in P_*pdaC*_ *-lux* (top), P_*iseA*_ *-lux* (middle), and P_*yocH*_ *-lux* (bottom) reporters in a Δ*prkC* P_*spac*_-*prkC* background grown in the presence of 0 and 10 μM IPTG. Expression in transition and stationary phases was compared to WT (gray), and Δ*prkC* (light blue) backgrounds.

We tested whether inducible heterologous PrkC expression complements a Δ*prkC* mutation specifically in stationary phase by comparing the expression of the WalR reporters in strains carrying an inducible copy of *prkC* (P_*spac*_-*prkC*) to their expression in WT and Δ*prkC* backgrounds (Fig 3). To confirm that these results represent systematic changes in expression from transition phase through early stationary phase (OD_600_~0.3–0.5), we also compared the normalized luminescence as a function of OD_600_ for each strain and IPTG concentration (S2 Fig). Without the addition of the inducer IPTG, the strain to test complementation, Δ*prkC* P_*spac*_-*prkC*, appears similar to Δ*prkC* for all three promoters (compare two lightest shades of blue, Figs 3 and S2). Under these conditions, the small amount of “leaky” expression without inducer is sufficient to complement expression of P_*yocH*_, and nearly complement P_*iseA*_ (compare gray and lightest shade of blue, Figs 3 and S2). At 10 μM IPTG, P_*spac*_-*prkC* complements the Δ*prkC* deletion for the expression of P_*pdaC*_, and P_*yocH*_ and P_*iseA*_ also display similar expression to the wild type (compare gray and blue, Figs 3 and S2). For each promoter, the effect of increasing inducer concentration was consistent with increasing WalR activity specifically in stationary phase: increased repression of *pdaC* and *iseA*, and increased activation of *yocH*. Although Δ*prkC* P_*spac*_-*prkC* was grown in the continuous presence of inducer, changes in gene expression for *pdaC*, *iseA*, and *yocH* did not appear until early stationary phase, consistent with the previous observation that the PrkC-dependent effect on the WalR regulon is growth phase specific (Figs 1 and 2). Thus, a low level of *prkC* expression *in trans* is sufficient to complement expression of *pdaC*, *iseA*, and *yocH* in stationary phase.

### PrkC overexpression resembles Δ*prpC* phenotypes

**Fig 4:**
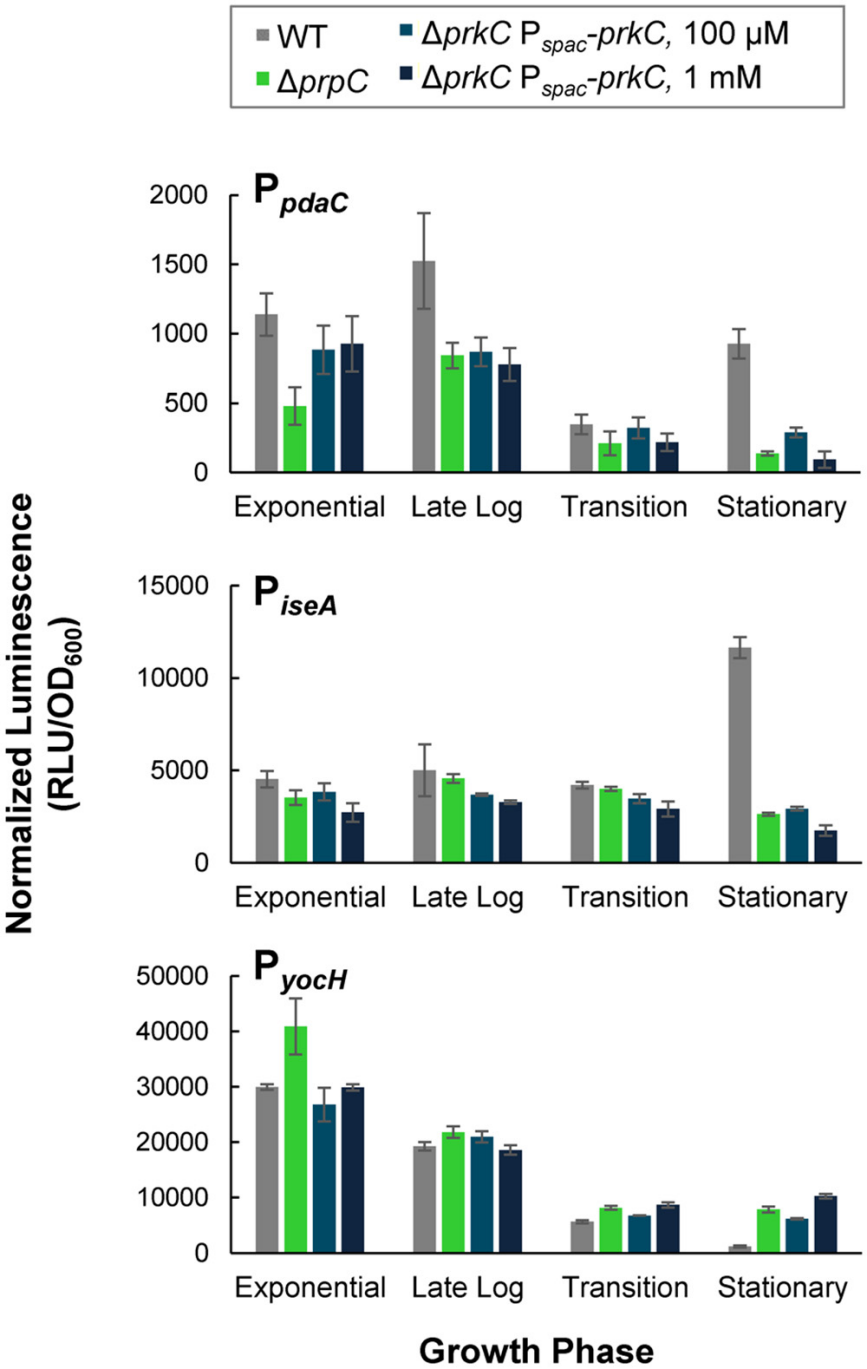
PrkC overexpression phenocopies Δ*prpC* effects on expression of WalR regulon genes. Normalized luminescence at characteristic growth phases of P_*pdaC*_ *-lux* (top), P_*iseA*_ *-lux* (middle), and P_*yocH*_ *-lux* (bottom) was measured in WT (gray), and Δ*prpC* (green) backgrounds and in a Δ*prkC* P_*spac*_-*prkC* background in the presence of 100 μM (medium blue) and 1 mM IPTG (dark blue).

The eSTK *prkC* is co-transcribed with its cognate eSTP *prpC*. Previous work suggested that the kinase activity of PrkC and the phosphatase activity of PrpC have opposing modes of action on *B*. *subtilis* physiology. Specifically, mutants in *prkC* and *prpC* have opposing stationary phase lysis phenotypes ([30] and Fig 1B) and overexpression of PrpC was shown to prevent PrkC mediated induction of *yocH* [15]. Consistent with these observations, *prkC* and *prpC* mutations have opposing effects on the expression of the WalR regulon genes *yocH*, *iseA*, and *pdaC* in stationary phase (Figs 1 and 2). We therefore sought to determine if the expression phenotypes for WalR regulon genes in a Δ*prpC* mutant background reflect high net PrkC kinase activity. To test this, we overexpressed *prkC* from P_*spac*_-*prkC* (in a strain lacking endogenous *prkC*) using higher levels of IPTG induction than were necessary to complement the Δ*prkC* mutation (Fig 3). Higher levels of induction (100 μM or 1 mM IPTG) of P_*spac*_-*prkC* caused expression of P_*yocH*_ to increase and expression of P_*iseA*_ and P_*pdaC*_ to decrease (Fig 4), consistent with further increases in WalR activity. These effects are similar to those observed in the presence of a Δ*prpC* mutation (green) suggesting that kinase overexpression overwhelms the phosphatase activity under these conditions (Fig 4). This further suggests that the Δ*prpC* expression phenotypes observed for *yocH*, *iseA*, and *pdaC* in stationary phase (Figs 1 and 2) are caused by high levels of PrkC-dependent WalR phosphorylation, generated by the absence of phosphatase activity. With 100 μM or 1 mM IPTG induction (shades of dark blue), the reporters in the Δ*prpC* mutant (green) background all appear similar (Fig 4), suggesting that the reporters in the Δ*prpC* background reflect the maximal kinase activity for that condition.

Although the *prpC-prkC* transcript is detected from exponential through stationary phase in LB [16], the heterologous overexpression of PrkC (Fig 4) produces only mild expression phenotypes prior to stationary phase. This observation, combined with the data indicating that PrkC-dependent transcriptional changes in the WalR regulon occur primarily in stationary phase (Figs 1 and 2), suggests that these effects are not simply caused by changes in *prkC* expression.

### The eSTK PrkC phosphorylates WalR *in vitro*

**Fig 5:**
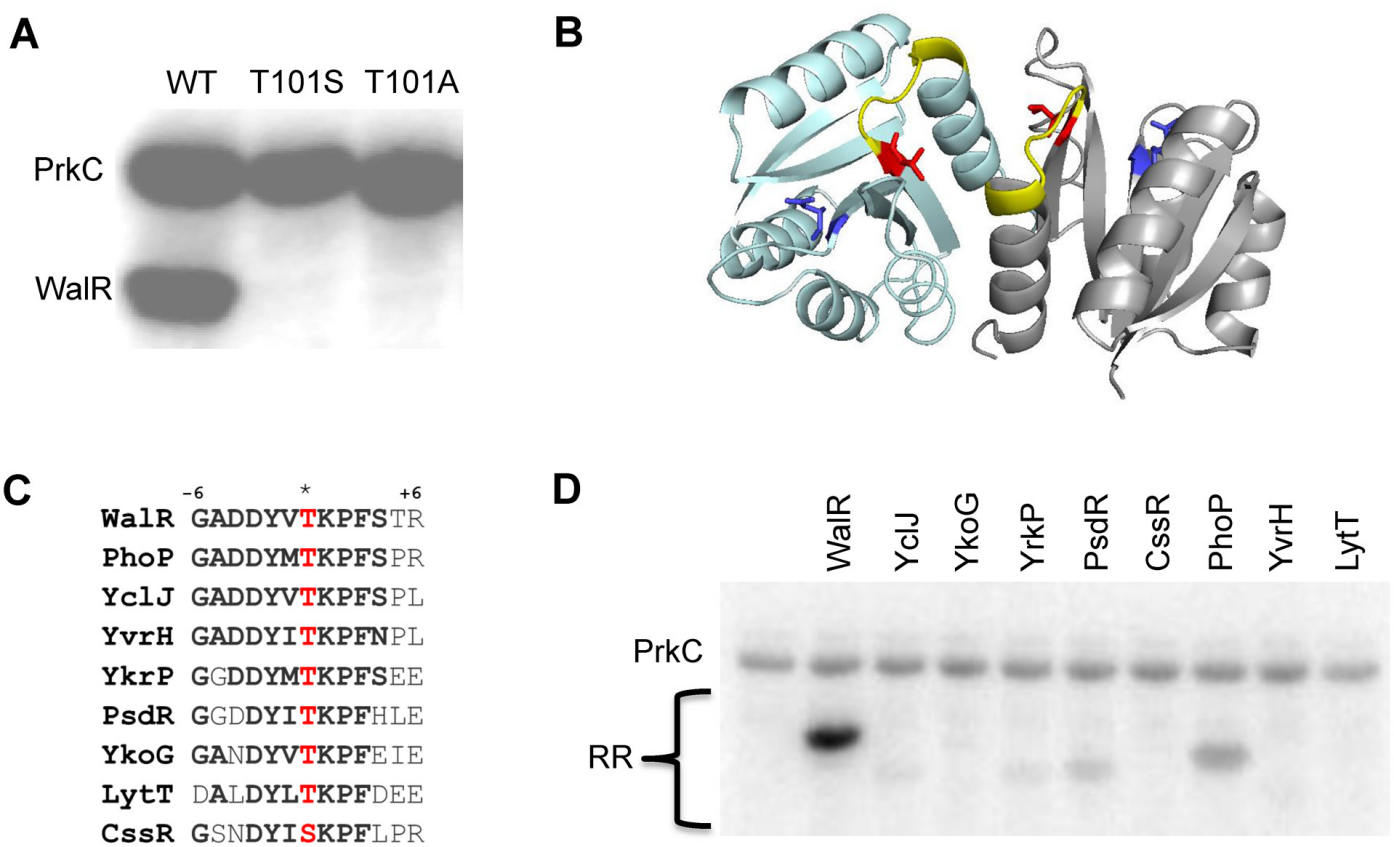
PrkC phosphorylates WalR Thr101 *in vitro*. **A)** PrkC transphosphorylates WalR but not a WalR T101S or a WalR T101A mutant protein in an *in vitro* kinase assay. 1 μM PrkC and 4 μM WalR, WalR T101S, or WalR T101A were incubated with [γ-^32^P]-ATP for 30 minutes at 37°C. **B)** WalR phosphosites highlighted on the WalR receiver domain dimer crystal structure (PDB ID: 2ZWM) with individual protomers shown in cyan and gray. Asp53, the site of WalK phosphorylation, is shown in blue and Thr101, the site of PrkC phosphorylation, is shown in red. Surface exposed residues from Lys102 through Arg107 (+6 position) are highlighted in yellow. **C)** Amino acid sequence conservation in the β5-α5 region of the receiver domain surrounding the conserved threonine or serine residue (*, red) for *B*. *subtilis* response regulators with a conserved threonine or serine residue at the homologous position to WalR Thr101. Response regulators are WalR, YclJ, YkoG, YrkP, PsdR, CssR, PhoP, YvrH, and LytT. **D)** PrkC has specificity for WalR *in vitro*. 1 μM PrkC and 4 μM of each response regulator in **(C)** was incubated at 37°C with [γ-^32^P]-ATP for 10 minutes or 30 minutes (S3 Fig).

As PrkC is membrane-bound and does not contain any predicted DNA binding domains, the observed co-regulation of three separate genes in the WalR regulon is likely to be indirect. One potential mechanism is a direct interaction of PrkC with WalR resulting in the phosphorylation of WalR on Ser or Thr residue(s). We examined this possibility by taking advantage of the previous observation that the cytoplasmic domain of PrkC autophosphorylates *in vitro* [34] and observed that PrkC robustly transphosphorylated WalR (Fig 5A). Mass spectrometry analysis of a PrkC-WalR kinase reaction performed with cold ATP suggested that WalR Thr101 was the only phosphorylated residue. To verify this interpretation, we generated mutant WalR proteins containing either a T101S or a T101A mutation and repeated the *in vitro* kinase assays. Strikingly, PrkC did not transphosphorylate either WalR T101S or WalR T101A mutant proteins, suggesting PrkC has a high degree of specificity for Thr101 (Fig 5A). WalR Thr101 is located at the dimerization interface of the receiver domain (Fig 5B, red).

### PrkC has specificity for WalR *in vitro*

Work with *M*. *tuberculosis* eSTKs revealed specificity for particular phosphoacceptors based on sequences surrounding the phosphosite [35]. A ClustalO alignment of the 34 *B*. *subtilis* response regulators identified eight response regulators with a Thr or Ser residue at the position homologous to WalR Thr101 (PhoP, YvrH, PsdR, YkoG, YclJ, YrkP, LytT, and CssR; Fig 5C). In all of these response regulators, the Ser or Thr residue is surrounded by well-conserved residues (Fig 5C, bold), including those located on the surface exposed loop from the +1 to +6 position (Fig 5B, yellow). However, despite this strong conservation, when we purified the eight response regulators and performed *in vitro* kinase assays with PrkC, we observed that PrkC much more robustly phosphorylated WalR than the other response regulators following 10’ incubation (Fig 5D). Increasing the incubation time of the reactions to 30’ did not increase the phosphorylation of any other substrates (S3A Fig). We verified that similar amounts of total protein were loaded for each response regulator (S3B Fig), and that none of the response regulators exhibited a background signal in the absence of PrkC (S3C Fig).

### WalR is phosphorylated on Thr101 *in vivo*

**Fig 6:**
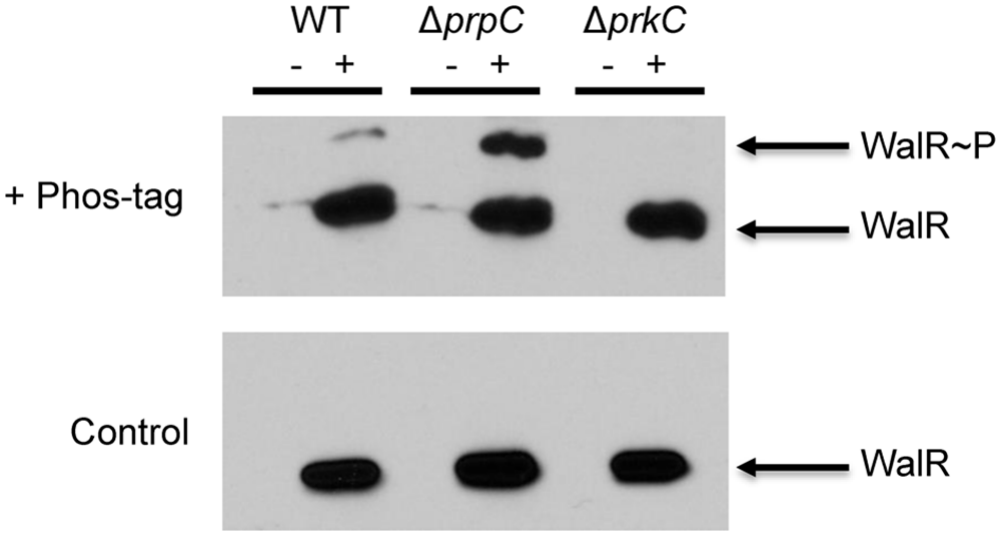
PrkC-dependent WalR phosphorylation is observed *in vivo*. Anti-FLAG western blots of WalR immunoprecipitated from WT, Δ*prpC*, and Δ*prkC* backgrounds expressing WalR-FLAG (‘+’ lanes). WT, Δ*prpC*, and Δ*prkC* strains lacking WalR-FLAG were used as controls (‘-‘ lanes). Top: SDS-PAGE supplemented with 37.5 μM Phos-tag results in a second band in WT and Δ*prpC* (Δphosphatase) backgrounds, but not in a Δ*prkC* (Δkinase). Bottom: In a control gel lacking Phos-tag, samples were identically loaded and run in parallel. To remove any residual heat labile Asp phosphorylation, samples were boiled prior to gel loading.

The ability of PrkC to affect the expression of WalR-dependent genes and to phosphorylate WalR *in vitro* on Thr101 suggests that this modification occurs *in vivo*. To detect WalR Thr phosphorylation *in vivo*, we immunoprecipitated FLAG-tagged WalR from OD_600_ matched stationary phase LB cultures of wild type (WT), Δ*prpC*, and Δ*prkC* strains. As a control for non-specific immunoprecipitation of proteins, strains lacking a FLAG-tagged protein were also used. Each sample was run on a gel cast with Phos-tag acrylamide, allowing for the separation of phosphorylated proteins by SDS-PAGE (Fig 6, top). To determine if any shifted bands observed were dependent on Phos-tag, and therefore indicative of phosphorylation, a control gel without Phos-tag was run in parallel (Fig 6, bottom). Since Thr phosphorylations are relatively heat stable compared to Asp phosphorylations, samples were boiled prior to gel loading. Visualized by subsequent Western blotting for the FLAG-tagged protein, a Phos-tag-dependent second band was observed in the WT sample, was significantly enhanced in the Δ*prpC* sample, but did not appear in the Δ*prkC* sample (Fig 6, top). This result indicated the presence of a heat stable PrkC-dependent WalR phosphorylation *in vivo*.

To verify that this PrkC-dependent WalR phosphorylation was on the same residue observed *in vitro*, mass spectrometry was performed on WalR-FLAG immunoprecipitated from Δ*prpC* cells. In two separate experiments, phospho-peptides containing Thr101 were found by mass spectrometry, providing further evidence that this modification occurs *in vivo* (S4 Fig). To confirm that the experimentally observed phospho-peptides and unmodified peptides had masses that are consistent with the WalR Thr101 region, the experimentally determined masses were compared to both the theoretically predicted masses and the masses observed from synthetic peptides (S4 Fig).

### WalR Thr101 is required for the PrkC-dependent effect on *yocH* expression

**Fig 7:**
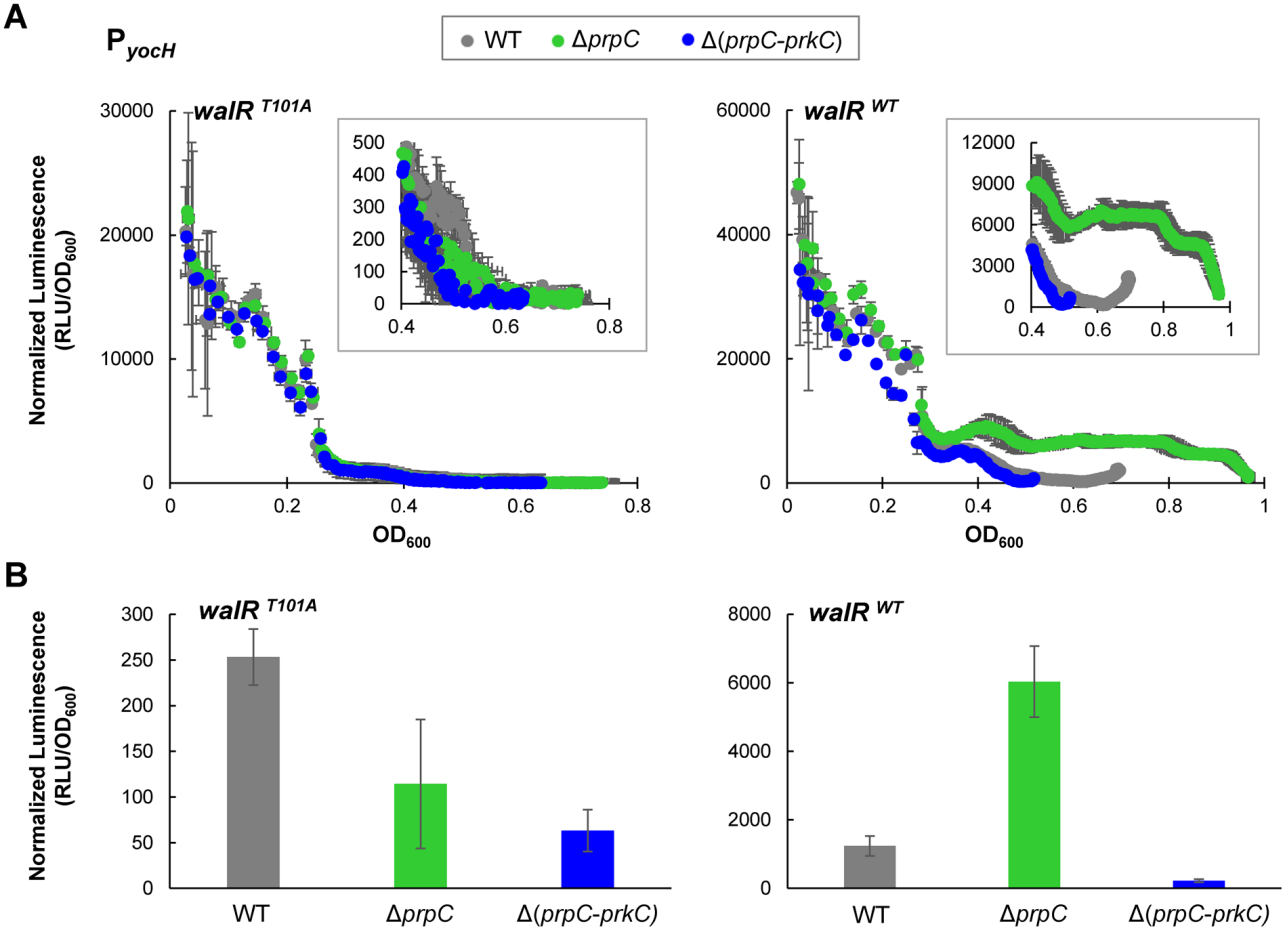
*yocH* does not show PrkC-dependent activation in a WalR T101A mutant. **A)** Parallel experiments measuring PrkC-dependent *yocH* activation in *walR* ^*T101A*^ point mutant (left) and *walR* ^*WT*^ strains (right). Normalized luminescence from a P_*yocH*_ *-lux* transcriptional reporter is plotted as a function of OD_600_ from exponential phase through the maximum OD_600_ reached for each strain. In both *walR* ^*T101A*^ and *walR* ^*WT*^ backgrounds, *yocH* is highly expressed in log phase. Insets: Detail of stationary phase expression of *yocH* in WT, Δ*prpC* and Δ(*prpC-prkC*) in *walR* ^*T101A*^ (left) and *walR* ^*WT*^ (right) backgrounds. **B)** Normalized luminescence of P_*yocH*_ *-lux* reporters in stationary phase (OD_600_~0.5) for *walR* ^*T101A*^ (left) and *walR* ^*WT*^ strains (right). In a *walR* ^*T101A*^ background, no significant difference is observed between Δ*prpC* and Δ(*prpC-prkC*) in stationary phase, whereas a >20 fold change is observed between Δ*prpC* and Δ(*prpC-prkC*) in a *walR* ^*WT*^ background (right).

PrkC-dependent WalR Thr101 phosphorylation was observed *in vivo*, suggesting that WalR Thr101 phosphorylation was responsible for the PrkC-dependent gene expression phenotypes observed for *yocH* (Fig 1). Therefore, we generated a strain containing a *walR* T101A mutation at the endogenous locus. Since the WalR T101A mutant may exhibit PrkC independent changes in expression compared to wild type WalR, direct comparison of *yocH* expression between *waR* ^*T101A*^ and *walR* ^*WT*^ backgrounds cannot be performed. Therefore, we generated P_*yocH*_ reporter strains in the *walR* ^*T101A*^ background in otherwise WT, Δ*prpC*, and Δ(*prpC-prkC*) backgrounds to test for PrkC dependence. In parallel, we measured the expression of *yocH* using normalized luminescence as a function of OD_600_ in both *walR* ^*T101A*^ and *walR* ^*WT*^ backgrounds (Fig 7A). Log phase *yocH* expression was comparable between *walR* ^*WT*^ and *walR* ^*T101A*^, indicating that the *walR* ^*T101A*^ mutant is functional at the *yocH* promoter *in vivo* (Fig 7A, compare OD_600_<0.3, left and right). Strikingly, in the *walR* ^*T101A*^ background, PrkC-dependent *yocH* activation in stationary phase was lost, with the WT, Δ*prpC* and Δ(*prpC-prkC*) backgrounds all showing similar expression profiles (Fig 7A, left). This is in contrast to the *walR* ^*WT*^ background, particularly in stationary phase (OD_600_>0.4), where the strongest PrkC-dependent activation is observed in a *walR* ^*WT*^ background (Fig 7A, right inset). At the same stationary phase OD_600_ used in Figs 1–4, we found that in the *walR* ^*WT*^ background, a >20 fold difference is observed between the Δ*prpC* and Δ(*prpC-prkC*) backgrounds, whereas no significant difference is observed in the *walR* ^*T101A*^ mutant background (Fig 7B). In the 40 min prior to OD_600_~0.5 (‘Stationary Phase’) we observed comparable growth rates for WT, Δ*prpC*, Δ*prkC*, Δ(*prpC-prkC*) in both *walR* ^*WT*^ (S5 Fig, top) and *walR* ^*T101A*^ (S5 Fig, bottom) backgrounds.

### PrkC-dependent repression of *iseA* and *pdaC* is reduced in a WalR T101A mutant

**Fig 8:**
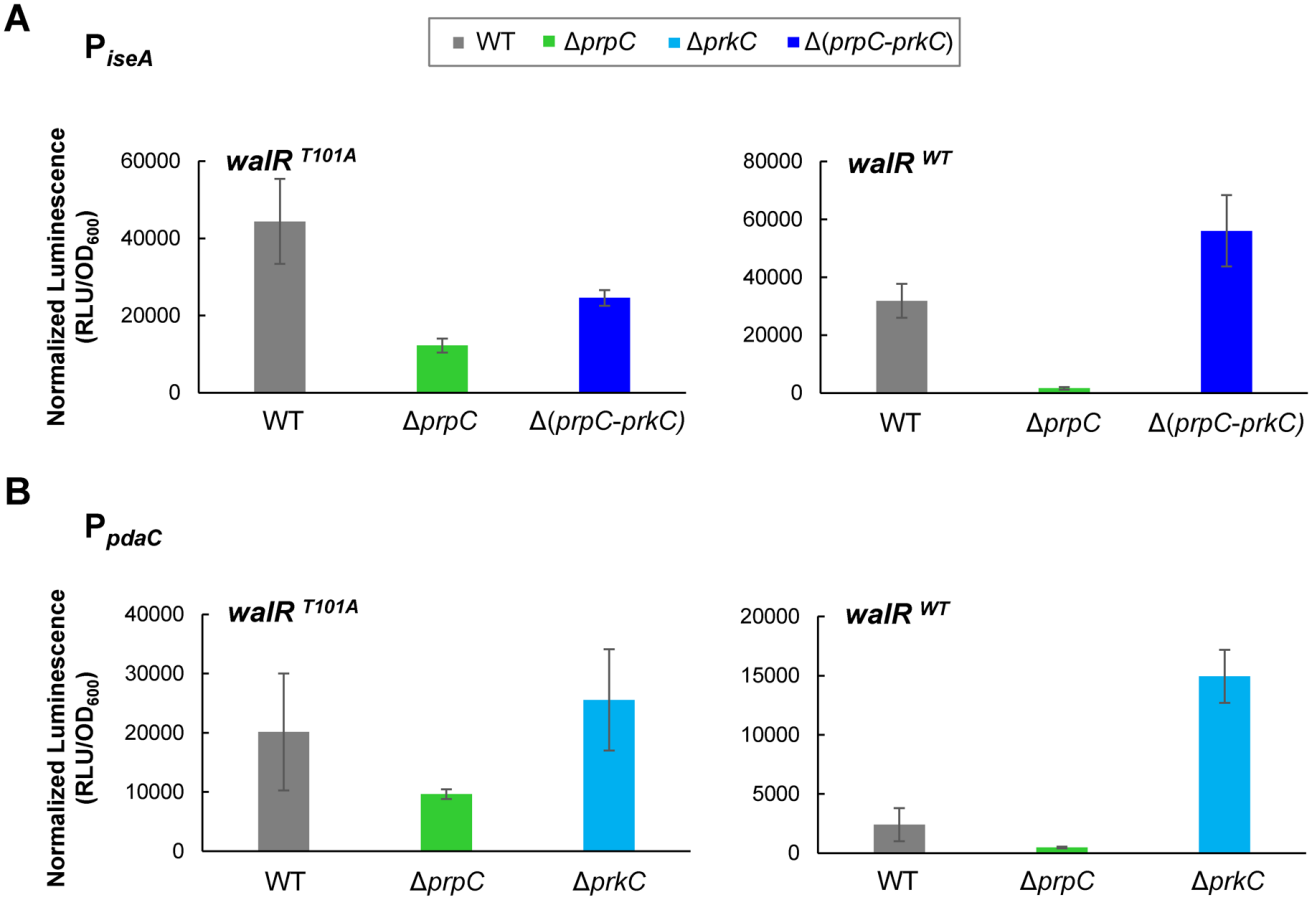
*iseA* and *pdaC* show reduced PrkC-dependent repression in a *walR* ^*T101A*^ mutant background. Normalized luminescence of P_*iseA*_ *-lux* **(A)** and P_*pdaC*_ *-lux* **(B)** reporters in stationary phase (OD_600_~0.5) in *walR* ^*T101A*^ (left) and *walR* ^*WT*^ strains (right). For both *iseA* and *pdaC*, repression was reduced >10 fold between Δ*prpC* and Δ*prkC* in the *walR* ^*T101A*^ mutant compared to the *walR* ^*WT*^ background.

To confirm that WalR Thr101 is also required for the PrkC-dependent repression of *iseA* and *pdaC*, we performed experiments similar to those described above for *yocH*. Log phase expression of *iseA* and *pdaC* in *walR* ^*T101A*^ and *walR* ^*WT*^ backgrounds was comparable, suggesting that WalR T101A is also functional at these promoters as a repressor (S6 Fig). Whereas both P_*iseA*_ and P_*pdaC*_ show strong PrkC-dependent repression in the *walR* ^*WT*^ background in stationary phase, in the *walR* ^*T101A*^ background, the repression is at least ~10 fold relieved between Δ*prpC* and Δ*prkC* (Fig 8). Consistent with a loss of PrkC-dependent activity at these promoters in stationary phase, the loss of repression in the *walR* ^*T101A*^ mutant is primarily due to the reduction of repression in the Δ*prpC* background, not to an increase in expression in a Δ*prkC* background. The residual-PrkC dependent effect on these promoters may be due to additional PrkC activity not dependent on WalR Thr101 phosphorylation that is sensed by WalK (S6 Fig). As *iseA* and *pdaC* were identified as the two sites in the *B*. *subtilis* chromosome with the highest WalR occupancy [25], it is possible that they are more sensitive than *yocH* to WalRK activity.

## Discussion

**Fig 9:**
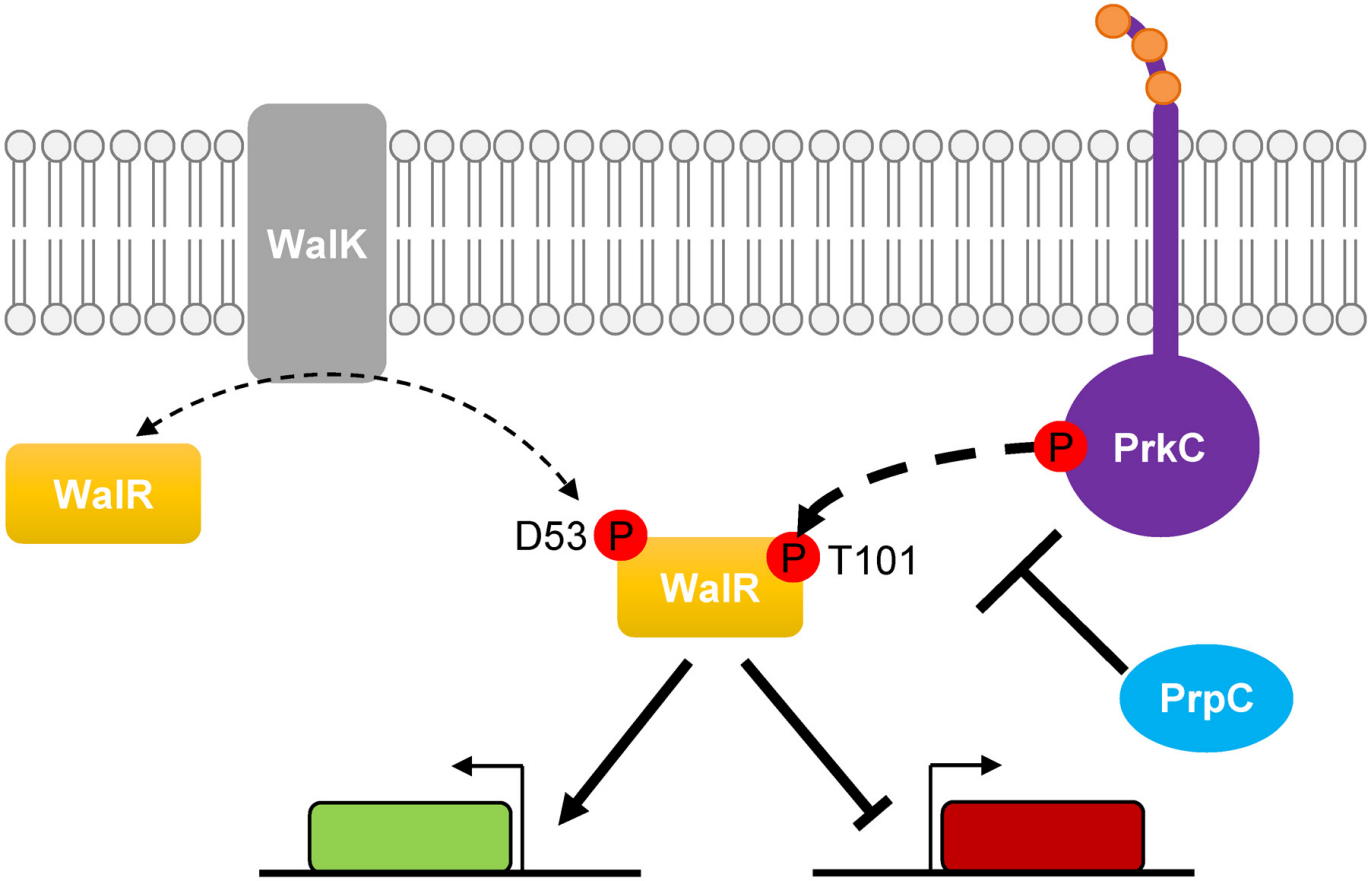
Model of PrkC dependent WalR phosphorylation. The response regulator WalR is phosphorylated on Asp53 by the histidine kinase WalK, leading to activation and repression of genes in the WalR regulon. WalR is also phosphorylated by the PASTA-domain-containing eSTK PrkC on Thr101 (bold dashed arrow) leading to enhanced activation or repression of genes in the WalR regulon. The eSTP PrpC decreases the amount of WalR Thr101 phosphorylation.

Here, we provide the first known mechanism for PrkC-dependent gene regulation in *B*. *subtilis* by demonstrating that PrkC phosphorylates the response regulator WalR on Thr101 both *in vitro* and *in vivo*. Overall, this work supports a model (Fig 9) where PrkC, a PASTA-domain-containing eSTK, is activated and phosphorylates WalR at Thr101. This is supported by the *in vitro* data demonstrating direct phosphorylation of WalR Thr101 by PrkC (Fig 5), as well as the presence of PrkC-dependent phosphorylation observed at Thr101 *in vivo* (Figs 6 and S4). Transcriptional data from the WalR-dependent genes *yocH*, *iseA*, and *pdaC* (Figs 1,2, 7 and 8) demonstrates that this secondary phosphorylation increases both the activation and repression of WalR regulon genes (Fig 9, bottom). In Δ*prpC* strains, increases in WalR Thr101 phosphorylation are observed *in vivo*, and changes in expression of WalR-dependent genes is consistent with high levels of PrkC activity (Figs 4 and 6), demonstrating that PrpC and PrkC have opposing modes of action.

The effect of PrkC on the expression of the WalR regulon is most significant as growth slows in stationary phase (Figs 1 and 2). As continuous expression of *prkC* in *trans* does not cause changes in gene expression during rapid growth, the mild effect of PrkC on the WalR regulon in log phase is likely not due to lack of expression. Rather, it suggests that PrkC regulation of the WalRK system becomes significant as the activity of the WalRK system begins to decrease in stationary phase [31]. Thus, by acting as a second sensor for the system, PrkC can provide an input to increase WalR activity even as WalK activity decreases. Since both PrkC and WalK are hypothesized to sense cell wall-related processes, and both are highly conserved signaling systems in Gram-positive bacteria, this may suggest that they have separate sensory inputs *in vivo*, such as sensing different aspects or different steps in cell wall growth and metabolism. The PASTA domain of PrkC interacts *in vitro* with muropeptides [13], suggesting that it may directly sense some aspect of the cell wall. WalK localizes to septal regions, and it has been hypothesized that some aspect of cell wall synthesis during active division is required to keep WalK activity high [18,36–39]. Taken together, separate sensory inputs working through PrkC and WalK may regulate the expression of genes responsible for cell wall metabolism by phosphorylation of WalR on two different sites.

WalR Thr101, the site of PrkC phosphorylation both *in vitro* and *in vivo*, is in the receiver domain at the α4-β5-α5 dimerization interface (Fig 5B). This region is important for both activity and regulation of protein-protein interactions in OmpR family response regulators such as WalR [40,41]. In response regulators of this type, this region has been shown to undergo important conformational changes when Asp phosphorylation occurs [42]. Therefore WalR Thr101 phosphorylation may impact the structural conformation of WalR in complex ways, including by altering the mechanism by which WalK-mediated Asp phosphorylation activates WalR. However, the gene expression data presented here suggests that the net effect of WalR Thr101 phosphorylation is consistent with an increase in WalR activity.

The high degree of sequence conservation between *B*. *subtilis* response regulators with conserved Ser or Thr residues in the receiver domain (Fig 5C) suggests that the specificity of PrkC for WalR is mediated by a region which is not conserved. Previous work dissecting the *in vitro* specificity of six different eSTKs in *M*. *tuberculosis* suggested that phosphorylation is enhanced with highly hydrophobic residues at the +3 and +5 positions surrounding the phosphosite [35]. Given that this region is surface exposed and the +3 site is conserved among the *B*. *subtilis* response regulators, the residue at the +5 position may govern specificity. This possibility is consistent with data that residues not directly adjacent to the phosphorylation site can affect the specific phosphorylation of response regulators by histidine kinases [43,44].

Other PrkC substrates including EF-Tu, EF-G, CpgA, YezB, YkwC, YvcK, and several metabolic enzymes [14,45–49] have been reported. However, evidence suggesting that PrkC-dependent phosphorylation of a transcription factor is important for gene regulation is relatively scarce. Recently, the transcription factor AbrB was found to be phosphorylated *in vitro* on S86 by PrkC and the two other *B*. *subtilis* eSTKs YabT and PrkD, resulting in a decrease in AbrB DNA binding activity [50]. However, AbrB phosphorylation *in vivo* was dependent on the presence of all three eSTKs, making the precise signal sensed and the dominant kinase responsible for physiologically relevant phosphorylation unclear.

Numerous examples of eSTK-dependent phosphorylation of transcription factors, including response regulators, have been reported in other Gram-positive bacteria (for recent reviews, see [2] and [8]). Although most of these examples are derived from *in vitro* observations, *in vivo* phosphorylation has been observed for both *S*. *pneumoniae* RR06 [51] and *S*. *pyogenes* CovR [52]. Examples in which specific *in vitro* phosphosites have been identified include *S*. *aureus* GraR [53] and VraR [54], *M*. *tuberculosis* DosR and Rv2175c [55–57], *S*. *pneumoniae* RitR [58] and RR06 [51], and Group A and Group B *Streptococci* CovR [52,59]. In some of these examples (e.g., RitR [58], GraR [53] and VraR [54]), the eSTK phosphorylates the response regulator in the DNA binding domain, suggesting that the effects on gene expression are caused by changing the affinity of the response regulator for its DNA binding sites. Alternatively, response regulators can be phosphorylated in the receiver domain (e.g., CovR [52,59], DosR [55], and VraR [54]). Of these, the most similar example to the WalR Thr101 phosphorylation we observed here is *S*. *aureus* VraR, which is phosphorylated *in vitro* on four residues including Thr106 located at the dimerization interface.

The essentiality of WalRK and its homologues in many low G+C Gram-positive bacteria, combined with its effect on virulence [60] and antibiotic resistance [61,62], has made mutations in the WalRK system in clinical pathogens of particular interest. Of note, single nucleotide substitutions within *walR* cause an increase in vancomycin resistance [63]. Whole genome sequencing of clinical isolates of VISA (vancomycin intermediate *S*. *aureus)* has identified WalR mutations (*walR T101S* and *walR T101R*) at the homologous Thr to the PrkC-WalR Thr phosphosite reported here [64]. This observation suggests that WalR Thr101 plays an important role in antibiotic resistance in *S*. *aureus*.

In conclusion, our characterization of PrkC-dependent phosphorylation of WalR helps to clarify the role of PrkC in stationary phase *B*. *subtilis* physiology and gene regulation. The intersection of the eSTK-eSTP pair PrkC-PrpC with the essential WalRK TCS system provides two sensory inputs to regulate the essential process of cell wall metabolism in *B*. *subtilis*. Parallels with closely related systems in Gram-positive pathogens suggest that this mechanism of regulation may be conserved and relevant to further understanding the role of eSTK phosphorylation of response regulators in Gram-positive physiology in the future.

## Materials and Methods

### Strains and strain construction

*Bacillus subtilis* strains were constructed by transformation into *B*. *subtilis* 168 *trpC2* (PB2) obtained from Chet Price [30] and its derivatives unless otherwise noted. Strains used in each figure are listed in S1 Table, and details of strain and plasmid construction are in S2, S3 and S4 Tables. *B*. *subtilis* strains were transformed with 5–10 μl of plasmid DNA or 1–2 μl of genomic DNA using the two-step method [65]. Genomic DNA was prepared using the Wizard Genomic DNA Purification Kit (Promega) according to the manufacturer’s instructions. Cultures were grown in LB (Lennox).

### Luminescence plate reader assays for growth curves and gene expression

Early log phase cultures were grown in LB inoculated from single colonies obtained from plates grown overnight at 37°C. The cultures were diluted 1:30 (150 μl final volume) into fresh LB in 96-well plates and grown at 37°C with continuous shaking for >20 h in a Tecan Infinite 200 plate reader. Measurements of luminescence and OD_600_ were taken at 5 min intervals. Media only and *lux*- controls were used for background subtraction for OD_600_ and luminescence respectively. All cultures were grown in triplicate. Note that OD_600_ values measured in the Tecan plate reader differ from the spectrophotometer by both a scale factor and an offset, making OD_600_~0.1 measurement in the plate reader correspond to OD_600_~0.4 in a standard laboratory spectrophotometer.

### Immunoprecipitation of WalR-FLAG

WalR-FLAG was immunoprecipitated from 25 ml (Phos-tag experiment) or 600 ml (mass spectrometry experiment) cultures at OD_600_~0.4 using anti-FLAG M2 magnetic beads (Sigma M8823) according to the manufacturer’s instructions with the following modifications. Initial lysis to break down the cell wall was performed in 50 mM Tris pH 7.5, 150 mM NaCl, 0.1% NP-40, 5 μg/ml DNaseI, 3 mM MgCl_2_, 1x Halt phosphatase inhibitor cocktail (Pierce), 1 mM PMSF, and 1 mg/ml lysozyme. After 20 min of lysis on ice with periodic vortexing, cell pellets were spun down and resuspended in lysis buffer without lysozyme and applied to 0.1 mm silica beads (BioSpec, 11079101z) in pre-chilled screw cap tubes. Cells were lysed using a FastPrep-24 5G instrument (MP Biomedicals) using 4 runs of 6.5 m/s for 40 seconds with samples placed on ice for 3 min between each run. Lysates were cleared by centrifugation at 19,000 x g for 20 min at 4°C. Elutions from the anti-FLAG M2 magnetic beads were performed competitively by addition of the 3x-FLAG peptide (Sigma F4799).

### Separation and visualization of phosphorylated WalR by Phos-tag acrylamide

10% resolving gels for SDS-PAGE were cast with ±37.5 μM Phos-tag acrylamide (Wako, AAL-107, 304–93521), and 5% stacking gels were prepared as per the manufacturer’s instructions. Prior to gel loading, 35 μL of each sample was boiled at 100°C for 15 seconds to remove any residual heat labile Asp phosphorylation. Approximately 30 ng of WalR-FLAG was loaded per lane. Gels were run and transferred as recommended by the manufacturer with the following modifications: SDS-PAGE gels were loaded and run in parallel on a Bio-Rad mini-protean gel apparatus at constant voltage (150 V) for approximately 100 min (an additional ~20 minutes after the dye front ran off the gel) at 4°C. Gels were fixed twice for 15 min in transfer buffer supplemented with 5 mM EDTA to remove Mn^2+^ from the gel, followed by a 10 min incubation in transfer buffer. Transfer to PVDF membranes was performed using a wet tank method (Bio-Rad Mini Trans-Blot) at a constant 350 mA for 80 min. Subsequent Western blotting was performed using standard protocols with an anti-FLAG-Peroxidase antibody (Sigma-Aldrich s#SAB4200119).

### *In vitro* kinase assays with PrkC

PrkC kinase reactions were performed in kinase buffer (50 mM Tris-HCl pH 7.5, 50 mM KCl, 10mM MgCl_2_, and 0.5 mM DTT). All reactions were performed in the presence of 400 μM cold ATP and 2 μCi (~ 20 nM) of γ-^32^P-ATP. The eSTK was used at a final concentration of 1 μM and substrates were added at a final concentration of 4 μM. Reactions were incubated at 37°C for 30 min unless otherwise indicated. 5X SDS-PAGE buffer was added to the reactions and subsequently boiled at 95°C for 5 min. Samples were run on a 12% SDS-PAGE gel and dried for 30 min at 80°C in a gel dryer. Dried gels were exposed to a phosphoscreen and visualized using a Typhoon Scanner (GE Healthcare).

Additional Materials and Methods, including protein purification and mass spectrometry for the detection of WalR phosphorylation both *in vitro* and *in vivo*, can be found in S1 Text.

## Supporting Information

S1 Fig Relative expression of P_*yocH*_ in WT and Δ*prkC* backgrounds in stationary phase. P_*yocH*_ expression in the WT (gray) compared to Δ*prkC* (light blue) and Δ(*prpC-prkC*) (dark blue) strains. Data shown is the detail of Fig 1C, ‘Stationary Phase’.
(TIFF)

S2 Fig Inducible PrkC expression complements Δ*prkC* expression phenotypes for *pdaC*, *iseA*, *and yocH* from transition phase through stationary phase. WT, Δ*prkC*, and Δ*prkC* P_*spac*_-*prkC* backgrounds carrying P_*pdaC*_ *-lux* (top), P_*iseA*_ *-lux* (middle), and P_*yocH*_ *-lux* (bottom) reporters were grown in the presence of 0 or 10 μM IPTG as indicated. Normalized luminescence was measured and plotted as a function of OD_600_ for 5 minute intervals during continuous growth from transition phase (OD_600_~0.3) to the max OD_600_ reached in stationary phase. For P_*yocH*_, the WT and Δ*prkC* P_*spac*_-*prkC* 0 μM IPTG curves are similar (overlapping points) from OD_600_~0.3–0.65.
(TIFF)

S3 Fig PrkC displays specificity for WalR. **A)** PrkC displays specificity for WalR *in vitro*. 1 μM PrkC and 4 μM of each response regulator in Fig 5 (WalR, YclJ, YkoG, YrkP, PsdR, CssR, PhoP, YvrH, and LytT) were incubated for 30 min at 37°C with [γ-^32^P]-ATP. **B)** Gel containing equivalent amounts of protein from (A) stained for total protein with Coomassie Brilliant Blue. **C)** Radioactive kinase assay using same conditions as Fig 5D without the addition of PrkC. No signal is detected from the response regulators in the absence of the kinase.
(TIFF)

S4 Fig WalR is phosphorylated on Thr101 *in vivo*. Mass spectrometry analysis was performed on WalR-FLAG immunoprecipitated from a cell lysate of a Δ*prpC* strain (also used in Fig 6) that was collected in stationary phase (equivalent to OD_600_~0.5 in the plate reader). The peptide [K.DSEIDKVIGLEIGADDYVTKPFSTR.E] containing Thr101 (red) was identified as being phosphorylated. The overall peptide masses (Tables) for the unmodified (top) and modified (phosphorylated, bottom) forms of the peptide were consistent between theoretical, experimental, and synthetic peptides for this WalR fragment. The fragmentation of the unmodified (top) and modified (bottom) peptide is consistent with Thr101 phosphorylation of the modified peptide, with ions containing Thr101 exhibiting mass shifts consistent with phosphorylation observed in the modified peptide.
(TIFF)

S5 Fig Growth curves of WT, Δ*prpC*, Δ*prkC*, Δ(*prpC-prkC*) in *walR* ^*WT*^ and *walR* ^*T101A*^ backgrounds approaching OD_600_~0.5. OD_600_ measurements over the 40 min prior to the OD_600_~0.5 ‘Stationary Phase’ measurements for the experiment in Fig 7 for each genetic background: WT, Δ*prpC*, Δ*prkC*, Δ(*prpC-prkC*) in both WalR^*WT*^ (top) and WalR^*T101A*^ (bottom).
(TIFF)

S6 Fig PrkC-dependent stationary phase repression of P_*iseA*_ *-lux* and P_*pdaC*_ *-lux* is relieved in a *walR* ^*T101A*^ mutant background. Normalized luminescence plotted as a function of OD_600_ of a P_*iseA*_ *-lux* **(A)** or P_*pdaC*_ *-lux* **(B)** reporter in strains expressing either *walR* ^*T101A*^ (left) or a *walR* ^*WT*^ (right) in WT (gray), Δ*prpC* (green), or Δ*prkC* (blue) backgrounds. Measurements were taken at 5 min intervals during continuous growth in LB and are plotted through the max OD_600_ reached for each strain.
(TIFF)

S1 Table Strains used in figures. (PDF)

S2 Table Strains used in this study. (PDF)

S3 Table Plasmids used in this study. (PDF)

S4 Table Oligonucleotides used in this study. (PDF)

S1 Text Supplementary materials and methods. (PDF)
