# Supplementary figures and images for: The Eukaryotic-Like Ser/Thr Kinase PrkC Regulates the Essential WalRK Two-Component System in Bacillus subtilis
Source: PLoS Genet. 2015 Jun 23;11(6):e1005275. doi: 10.1371/journal.pgen.1005275 (PMC4478028; doi:10.1371/journal.pgen.1005275)

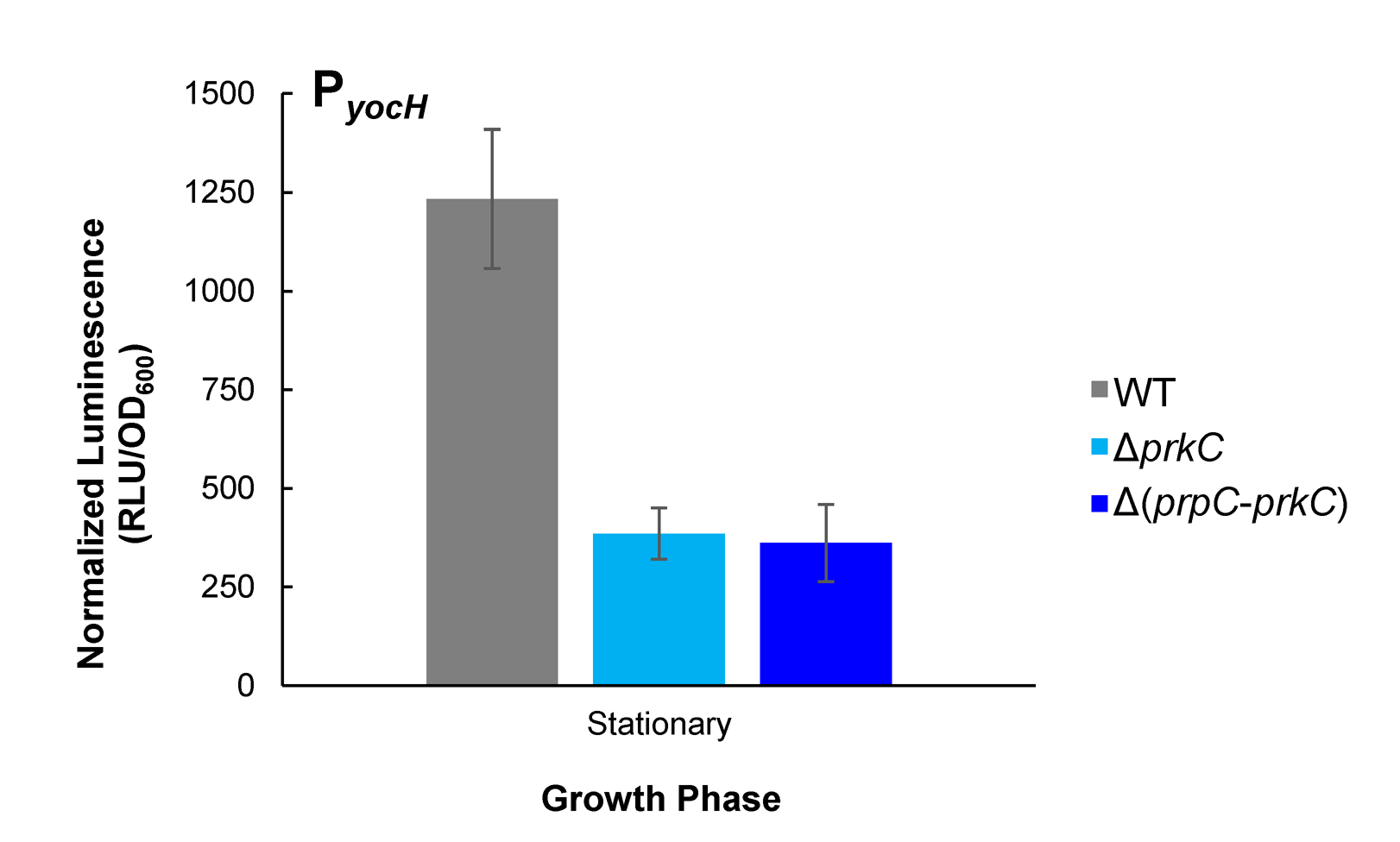

Supplement: S1 Fig — PyocH expression in the WT (gray) compared to ΔprkC (light blue) and Δ(prpC-prkC) (dark blue) strains. Data shown is the detail of Fig 1C, ‘Stationary Phase’. (TIFF) [file pgen.1005275.s001.tiff]

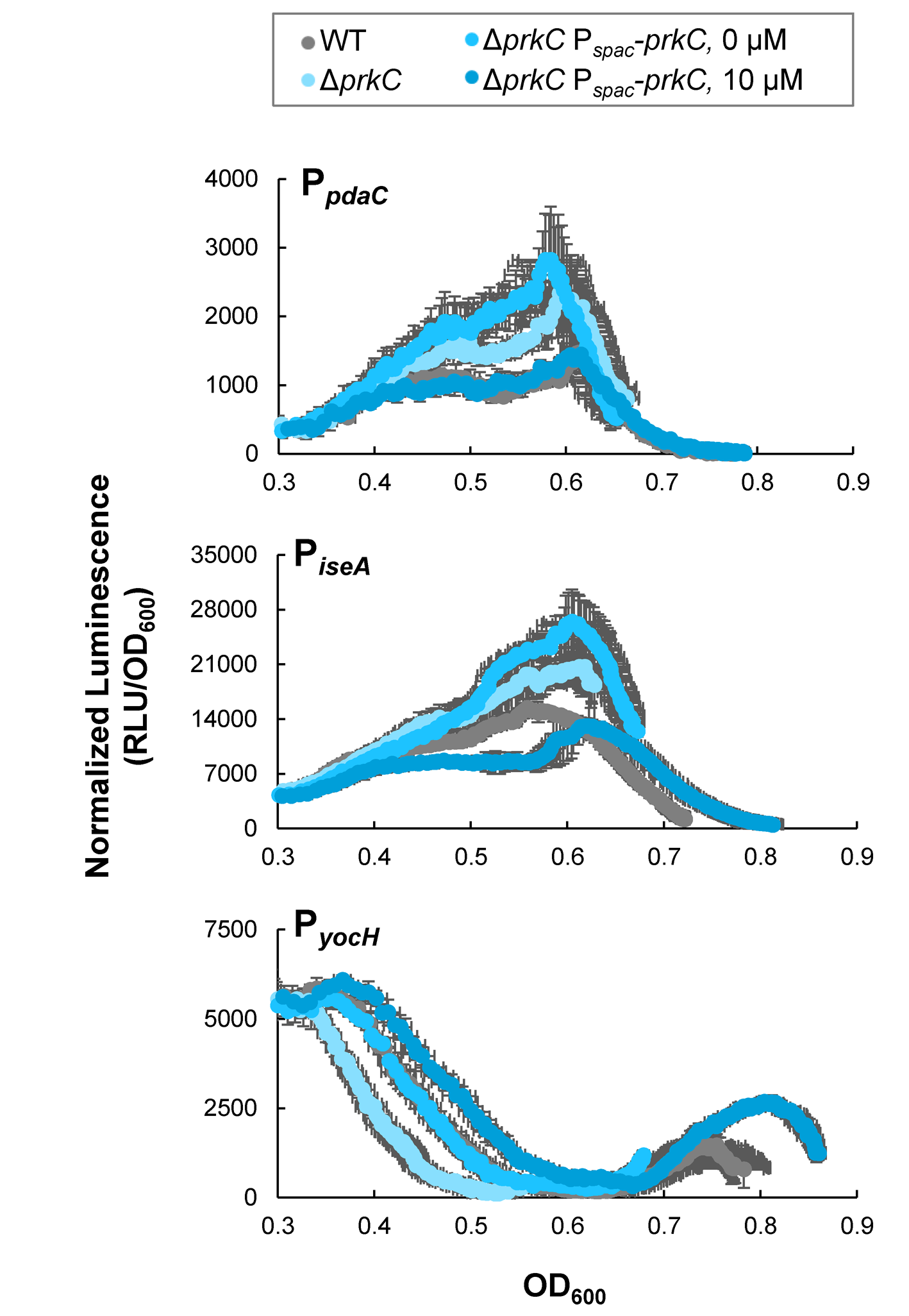

Supplement: S2 Fig — WT, ΔprkC, and ΔprkC Pspac-prkC backgrounds carrying PpdaC -lux (top), PiseA -lux (middle), and PyocH -lux (bottom) reporters were grown in the presence of 0 or 10 μM IPTG as indicated. Normalized luminescence was measured and plotted as a function of OD600 for 5 minute intervals during continuous growth from transition phase (OD600~0.3) to the max OD600 reached in stationary phase. For PyocH, the WT and ΔprkC Pspac-prkC 0 μM IPTG curves are similar (overlapping points) from OD600~0.3–0.65. (TIFF) [file pgen.1005275.s002.tiff]

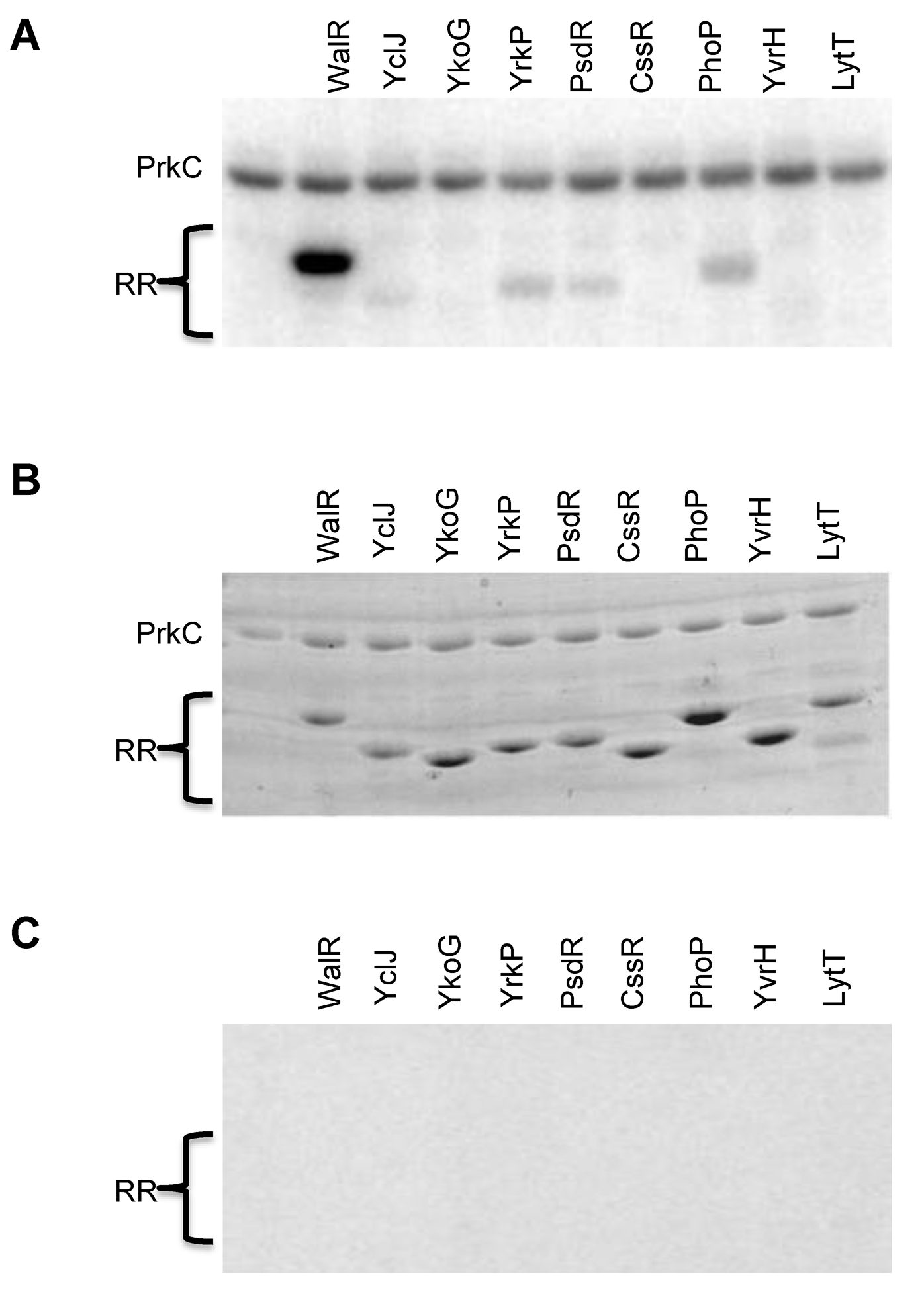

Supplement: S3 Fig — A) PrkC displays specificity for WalR in vitro. 1 μM PrkC and 4 μM of each response regulator in Fig 5 (WalR, YclJ, YkoG, YrkP, PsdR, CssR, PhoP, YvrH, and LytT) were incubated for 30 min at 37°C with [γ-32P]-ATP. B) Gel containing equivalent amounts of protein from (A) stained for total protein with Coomassie Brilliant Blue. C) Radioactive kinase assay using same conditions as Fig 5D without the addition of PrkC. No signal is detected from the response regulators in the absence of the kinase. (TIFF) [file pgen.1005275.s003.tiff]

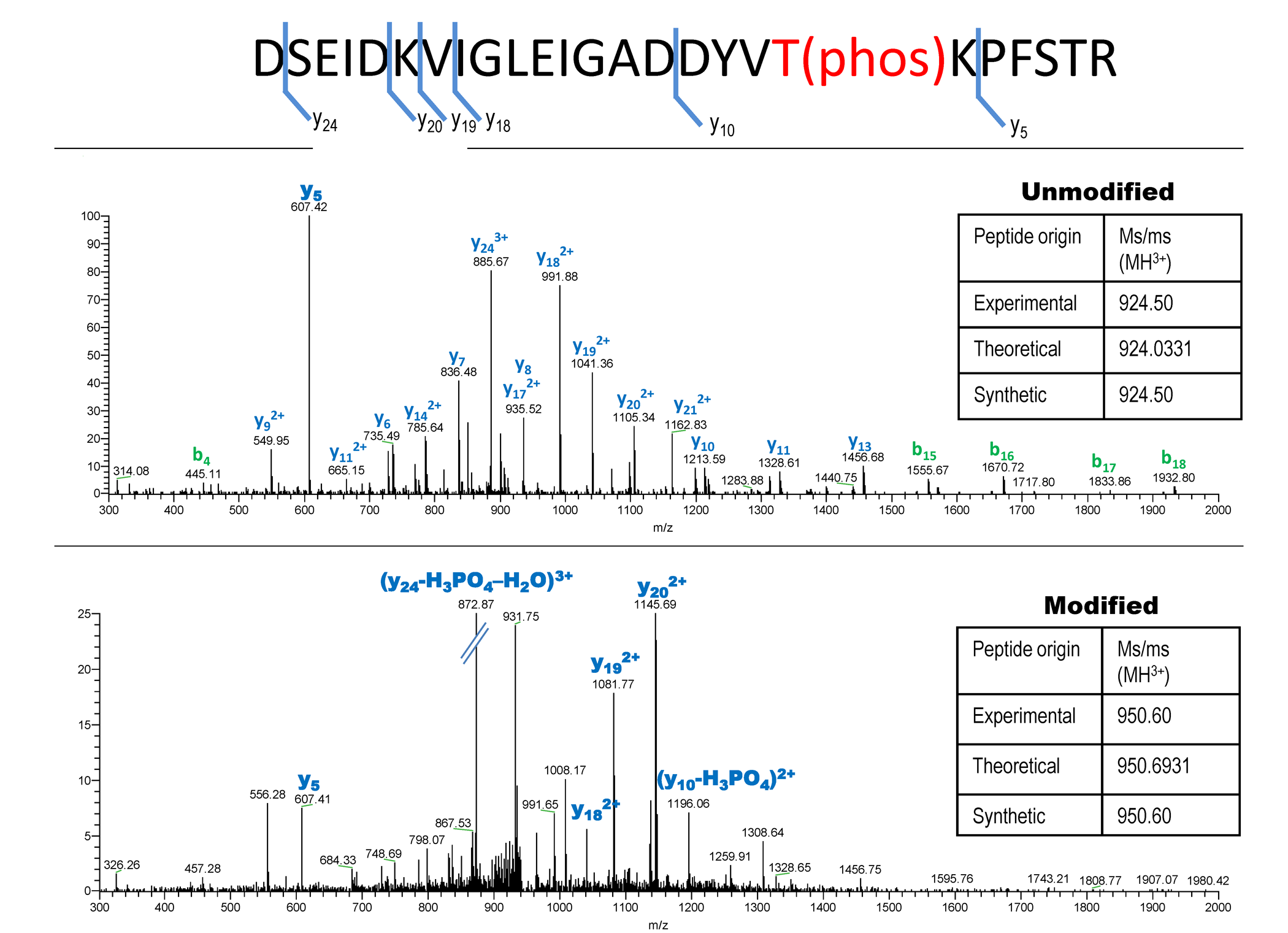

Supplement: S4 Fig — Mass spectrometry analysis was performed on WalR-FLAG immunoprecipitated from a cell lysate of a ΔprpC strain (also used in Fig 6) that was collected in stationary phase (equivalent to OD600~0.5 in the plate reader). The peptide [K.DSEIDKVIGLEIGADDYVTKPFSTR.E] containing Thr101 (red) was identified as being phosphorylated. The overall peptide masses (Tables) for the unmodified (top) and modified (phosphorylated, bottom) forms of the peptide were consistent between theoretical, experimental, and synthetic peptides for this WalR fragment. The fragmentation of the unmodified (top) and modified (bottom) peptide is consistent with Thr101 phosphorylation of the modified peptide, with ions containing Thr101 exhibiting mass shifts consistent with phosphorylation observed in the modified peptide. (TIFF) [file pgen.1005275.s004.tiff]

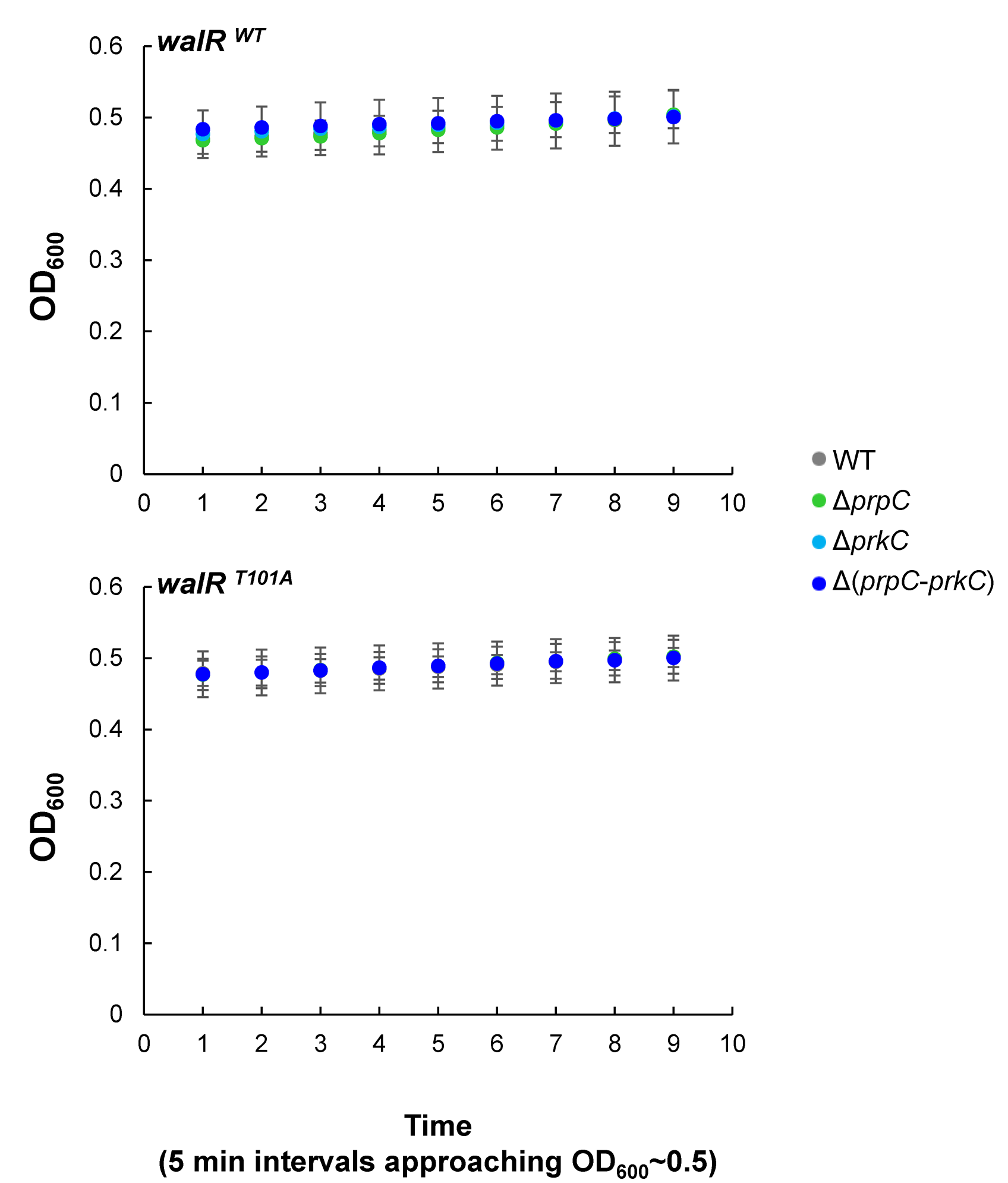

Supplement: S5 Fig — OD600 measurements over the 40 min prior to the OD600~0.5 ‘Stationary Phase’ measurements for the experiment in Fig 7 for each genetic background: WT, ΔprpC, ΔprkC, Δ(prpC-prkC) in both WalRWT (top) and WalRT101A (bottom). (TIFF) [file pgen.1005275.s005.tiff]

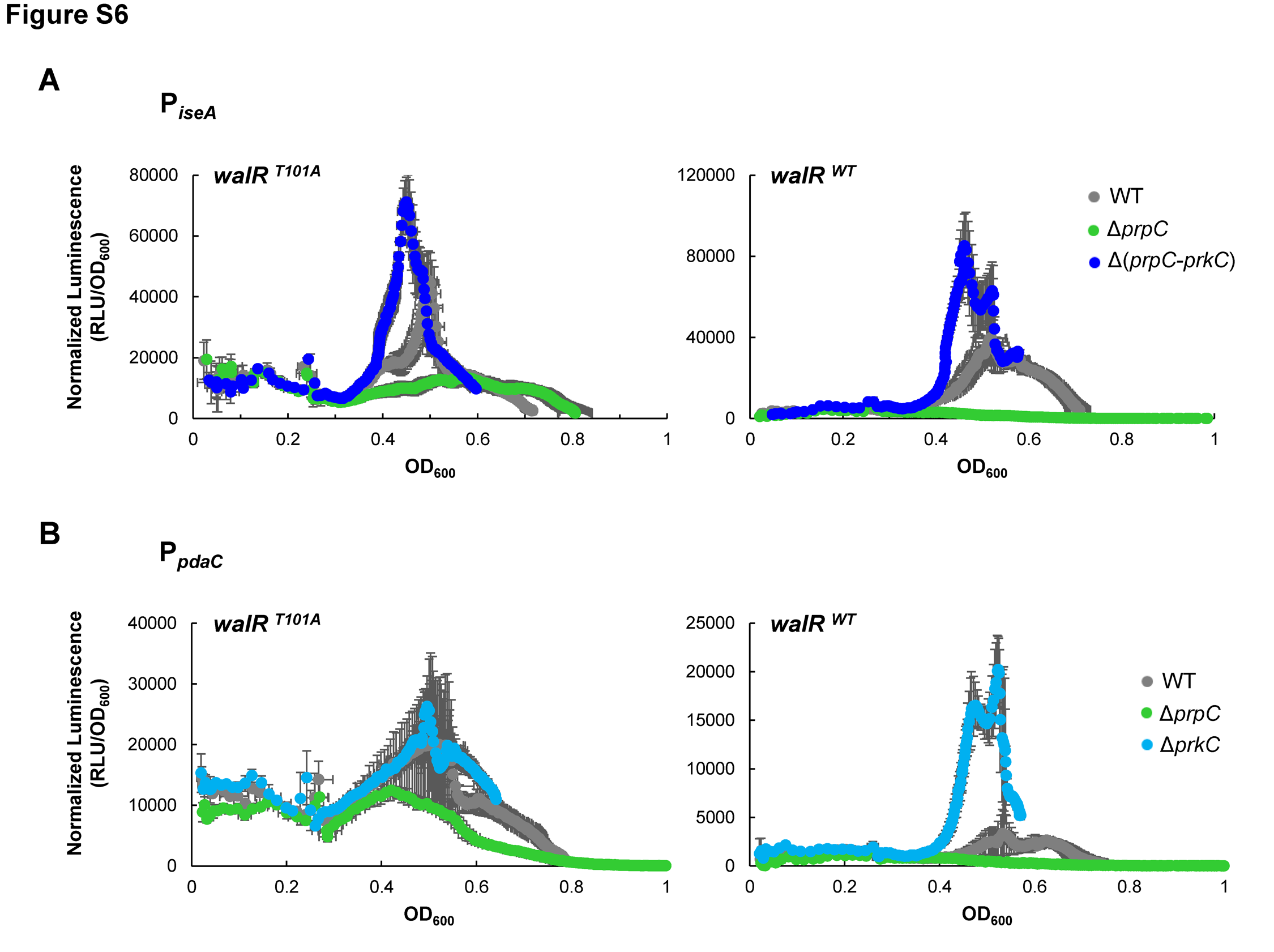

Supplement: S6 Fig — Normalized luminescence plotted as a function of OD600 of a PiseA -lux (A) or PpdaC -lux (B) reporter in strains expressing either walR T101A (left) or a walR WT (right) in WT (gray), ΔprpC (green), or ΔprkC (blue) backgrounds. Measurements were taken at 5 min intervals during continuous growth in LB and are plotted through the max OD600 reached for each strain. (TIFF) [file pgen.1005275.s006.tiff]
