# Supplementary material for: The Eukaryotic-Like Ser/Thr Kinase PrkC Regulates the Essential WalRK Two-Component System in Bacillus subtilis
Source: PLoS Genet. 2015 Jun 23;11(6):e1005275. doi: 10.1371/journal.pgen.1005275 (PMC4478028; doi:10.1371/journal.pgen.1005275)
Supplement: S1 Table — (PDF) [file pgen.1005275.s007.pdf]

S1 Table. Strains used for data in figures

Figure	Panel	Strains
**1**		ELB205, ELB211, ELB214, ELB217
**2**	A and B	P_{iseA}: ELB204, ELB210, ELB213, ELB216<br>P_{pdaC}: ELB203, ELB209, ELB212, ELB215
**3**		P_{iseA}: ELB204, ELB210, ELB213, ELB252<br>P_{pdaC}: ELB203, ELB209, ELB212, ELB251<br>P_{yocH}: ELB205, ELB211, ELB214, ELB253
**4**		Same as 3
**5**	A	JDE1582, JDE1591, JDE1713, JDE1708
	D	JDE1582, JDE1591, JDE1854, JDE1855, JDE1856, JDE1857, JDE1858, JDE1862, JDE1948, JDE1905
**6**		JDB3442, JDB3444, JDB3446, PB2, PB702, PB705
**7**	A and B	*walR*: ELB205, ELB211, ELB217<br>*walR T101A*: ELB243, ELB249, ELB299
**8**	A	*walR*: ELB204, ELB210, ELB216<br>*walR T101A*: ELB242, ELB254, ELB256
	B	*walR*: ELB203, ELB209, ELB212<br>*walR T101A*: ELB241, ELB231, ELB244
**S1**		ELB205, ELB214, ELB217
**S2**		Same as 5D
**S5**		Same as 7
**S6**	A	Same as 8A
	B	Same as 8B
