# Supplementary material for: The Eukaryotic-Like Ser/Thr Kinase PrkC Regulates the Essential WalRK Two-Component System in Bacillus subtilis
Source: PLoS Genet. 2015 Jun 23;11(6):e1005275. doi: 10.1371/journal.pgen.1005275 (PMC4478028; doi:10.1371/journal.pgen.1005275)
Supplement: S2 Table — (PDF) [file pgen.1005275.s008.pdf]

S2 Table: Strains Used in this Study

Strain	Genotype	Construction	Source
***B. subtilis* strains**			
168 *trpC2* (PB2)	168 *trpC2* (WT)		Lab stock [30]
PY79	Prototroph		Lab stock
PB702	PB2 Δ*prpC*		[30]
PB705	PB2 Δ*prkC*		[30]
PB722	PB2 Δ*prpC-prkC*		[30]
JDB3221	*purA-kan-walRT101A*	Integration of ligation of XbaI/SalI fragment of pLG225 with SalI/KpnI fragment of pLG232	This study
JDB3429	*trpC2* amyE::P$_{walR}$-*walR-FLAG spec*	Integration of pLG138 into PB2	This study
JDB3442	*trpC2 amyE*::P$_{walR}$-*walR-FLAG spec*, *walR*::*kan*	Integration of pLG91 into JDB3429	This study
JDB3444	*trpC2* Δ*prpC amyE*::P$_{walR}$-*walR-FLAG spec*, *walR*::*kan*	Transformations of JDB3442 into PB702	This study
JDB3446	*trpC2* Δ*prkC amyE*::P$_{walR}$-*walR-FLAG spec*, *walR*::*kan*	Transformations of JDB3442 into PB705	This study
JDB3735	*trpC2* Δ*prkC* P$_{spac}$-*prkC spec*	Integration of pLG393 into PB705	This study
**P$_{pdaC}$ Reporters**			
ELB203	*trpC2 sacA*::P$_{pdaC}$-*luxABCDE cm*	Integration of pEL130 into PB2	This study
ELB209	*trpC2* Δ*prpC sacA*::P$_{pdaC}$-*luxABCDE cm*	Transformation of ELB203 into PB702	This study
ELB212	*trpC2* Δ*prkC sacA*::P$_{pdaC}$-*luxABCDE cm*	Transformation of ELB203 into PB705	This study
ELB215	*trpC2* Δ*prpC-prkC sacA*::P$_{pdaC}$-*luxABCDE cm*	Transformation of ELB203 into PB722	This study
ELB241	*trpC2 sacA*::P$_{pdaC}$-*luxABCDE cm*, *purA-kan-walRT101A*	Transformation of JDB3221 into ELB203	This study
ELB231	*trpC2* Δ*prpC sacA*::P$_{pdaC}$-*luxABCDE cm*, *purA-kan-walRT101A*	Transformation of JDB3221 into ELB209	This study
ELB244	*trpC2* Δ*prkC sacA*::P$_{pdaC}$-*luxABCDE cm*, *purA-kan-walRT101A*	Transformation of JDB3221 into ELB212	This study
ELB251	*trpC2* Δ*prkC* P$_{spac}$-*prkC spec*, *sacA*::P$_{pdaC}$-*luxABCDE cm*	Transformation of ELB203 into JDB3735	This study
**P$_{iseA}$ Reporters**			
ELB204	*trpC2 sacA*::P$_{iseA}$-*luxABCDE cm*	Integration of pEL131 into PB2.	This study
ELB210	*trpC2* Δ*prpC sacA*::P$_{iseA}$-*luxABCDE*	Transformation of	This study

	cm	ELB204 into PB702	
ELB213	*trpC2* Δ*prkC sacA*::P$_{iseA}$-*luxABCDE cm*	Transformation of ELB204 into PB705	This study
ELB216	*trpC2* Δ*prpC-prkC sacA*::P$_{iseA}$-*luxABCDE cm*	Transformation of ELB204 into PB722	This study
ELB242	*trpC2 sacA*::P$_{iseA}$-*luxABCDE cm*, *purA-kan-walRT101A*	Transformation of JDB3221 into ELB204	This study
ELB252	*trpC2* Δ*prkC* P$_{spac}$-*prkC spec*, *sacA*::P$_{iseA}$-*luxABCDE cm*	Transformation of ELB204 into JDB3735	This study
ELB254	*trpC2* Δ*prpC sacA*::P$_{iseA}$-*luxABCDE cm*, *purA-kan-walRT101A*	Transformation of JDB3221 into ELB210	This study
ELB256	*trpC2* Δ*prpC-prkC sacA*::P$_{iseA}$-*luxABCDE cm*, *purA-kan-walRT101A*	Transformation of JDB3221 into ELB216	This study
**P$_{yocH}$ Reporters**			
ELB205	*trpC2 sacA*::P$_{yocH}$-*luxABCDE cm*	Integration of pEL132 into PB2	This study
ELB211	*trpC2* Δ*prpC sacA*::P$_{yocH}$-*luxABCDE cm*	Transformation of ELB205 into PB702	This study
ELB214	*trpC2* Δ*prkC sacA*::P$_{yocH}$-*luxABCDE cm*	Transformation of ELB205 into PB705	This study
ELB217	*trpC2* Δ*prpC-prkC sacA*::P$_{yocH}$-*luxABCDE cm*	Transformation of ELB205 into PB722	This study
ELB243	*trpC2 sacA*::P$_{yocH}$-*luxABCDE cm*, *purA-kan-walRT101A*	Transformation of JDB3221 into ELB205	This study
ELB249	*trpC2* Δ*prpC-prkC sacA*::P$_{yocH}$-*luxABCDE cm*, *purA-kan-walRT101A*	Transformation of JDB3221 into ELB217	This study
ELB253	*trpC2* Δ*prkC* P$_{spac}$-*prkC spec*, *sacA*::P$_{yocH}$-*luxABCDE cm*	Transformation of ELB205 into JDB3735	This study
ELB299	*trpC2* Δ*prpC sacA*::P$_{yocH}$-*luxABCDE cm*, *purA-kan-walRT101A*	Transformation of JDB3221 into ELB211	This study
**E. coli Strains**			
JDE1601	pLG43 (WalK-His$_6$)	Transformation into BL21(DE3)	This study
JDE1591	pLG44 (WalR-His$_6$)	"	This study
JDE1582	pSFP37(His$_6$-PrkC)	"	This study
JDE1708	pLG98 (WalR T101A-His$_6$)	"	This study
JDE1713	pLG103 (WalR T101S-His$_6$)	"	This study
JDE1854	pLG145 (YclJ-His$_6$)	"	This study
JDE1855	pLG146 (YkoG-His$_6$)	"	This study
JDE1856	pLG147 (YkrP-His$_6$)	"	This study

JDE1857	pLG148 (YvcP-His_6)	“	This study
JDE1858	pLG149 (CssR-His_6)	“	This study
JDE1862	pLG158 (PhoP-His_6)	“	This study
JDE1948	pLG178 (YvrH-His_6)	“	This study
JDE1905	pLG199 (LytT-His_6)	“	This study
