# Supplementary material for: The Eukaryotic-Like Ser/Thr Kinase PrkC Regulates the Essential WalRK Two-Component System in Bacillus subtilis
Source: PLoS Genet. 2015 Jun 23;11(6):e1005275. doi: 10.1371/journal.pgen.1005275 (PMC4478028; doi:10.1371/journal.pgen.1005275)
Supplement: S3 Table — (PDF) [file pgen.1005275.s009.pdf]

S3 Table: Plasmids used in this study

**Plasmid**			**Source**
**Stock Vectors**			
pDG780			[66]
pDG1662			[67]
pETDUET			Novagen
pSac-cm			[68]
pDR111			[69]
**Plasmid**	**Genotype**	**Construction**	**Source**
**For *B. subtilis* Integration**			
pEL101	pDG1662 P_{ywaC}-*luxABCDE*	Subcloning of the *luxABCDE* transcriptional reporter amplified from EB1385 [70] using PywaC-luxABCDE-u1 and PywaC-luxABCDE-l1 into pDG1662 digested with EcoRI/HindIII by Gibson assembly.	This study
pEL130	pSac-cm P_{pdaC}-*luxABCDE*	P_{pdaC}-*luxABCDE* cloned into pSac-cm by 3 way ligation. Amplified the *pdaC* (*yjeA*) promoter region from *Bs*.168 *trpC2* using BamHI-PyjeA-u1 and SalI-PyjeA-l1 and digested with BamHI/SalI. Ligated to pSac-cm digested with EcoRI/BamHI, and pEL101 EcoRI/SalI.	“
pEL131	pSac-cm P_{iseA}-*luxABCDE*	P_{iseA}-*luxABCDE* cloned into pSac-cm by 3 way ligation. Amplified *iseA* (*yoeB*) the promoter region from Bs.168 *trpC2* using BamHI-PyoeB-u1 and SalI-PyoeB-l1 and digested with BamHI/SalI. Ligated to pSac-cm digested with EcoRI/BamHI, and pEL101 EcoRI/SalI.	“
pEL132	pSac-cm P_{yocH}-*luxABCDE*	P_{yocH}-*luxABCDE* cloned into pSac-cm by 3 way ligation. Amplified the *yocH* promoter region using BamHI-PyocH-u1 and SalI-PyocH-l1 and digested with BamHI/SalI. Ligated to pSac-cm digested with EcoRI/BamHI, and pEL101	“

		EcoRI/SalI.	
pLG7	P_{walR}-*walR-FLAG*	P_{walR}-*walR-FLAG* amplified from chromosome using LG66/LG67, digested with EcoR1/BamH1 and ligated into pDG1662 digested with EcoR1 and BamH1	“
pLG91	*walR::kan*	*walR* deletion vector constructed by cloning homology to the region surrounding *walR* into pDG780. Homology was amplified using LG174/LG178 (EcoRI/BamHI) and LG179/LG177 (SalI/KpnI).	“
pLG138	P_{walR}-*walR-FLAG*	pLG7 digested with EcoR1/BamHI, and ligated to pDG1730 digested with EcoRI/BamHI.	“
pLG225	*walR* allelic replacement; kan^R	1kb upstream of *walRK* promoter amplified with LG420/LG421 digested with Xba1/BamH1 and ligated into pDG780	“
pLG232	*walR T101A* allelic replacement; kan^R	*walRK* promoter and *walR T101A*. Site directed mutagenesis by SOE using pLG231 as a template and oligos **LG422/**LG190 and **LG423/**LG189. The resulting product was digested with Sal1/ Kpn1 and ligated into pDG780.	“
pLG393	P_{spac}-*FLAG-prkC* spec; for integration at *amyE*	Full length *prkC* including optimized RBS amplified with LG836/837 and inserted into Nhe1-linearized pDR111 by Gibson Cloning.	“
**For *E. coli* expression**			
pLG43	T7-'*walK*-6his; amp^R	Cytoplasmic domain of *walK* amplified using LG129/LG134 and digested with Nde1/AatI and ligated into pETDUET	“
pLG44	T7-*walR*-6his; amp^R	*walR* amplified using LG130/LG135 digested with Nde1/Xho1 and ligated into pETDUET	“
pLG98	T7-*walR* (T101A)-6his; amp^R	*walR T101A* generated by SOE using pLG44 as a template and	“

		oligos **LG130**/LG189 and **LG135**/LG190. The resulting product was digested with Nde1/Xho1 and ligated into pETDUET	
pLG103	T7-*walR* (T101S)-6his; amp^R	*walR T101S* generated by SOE using pLG44 as a template and oligos **LG130**/LG225 and **LG135**/LG242. The resulting product was digested with Nde1/Xho1 and ligated into pETDUET	"
pLG145	T7-*yclJ*-6his; amp^R	*yclJ* amplified with LG298/LG299 and digested with Nde1/Xho1 and ligated into pETDUET	"
pLG146	T7-*ykoG*-6his; amp^R	*ykoG* amplified with LG300/LG301 and digested with Nco1/ Xho1 and ligated into pETDUET	"
pLG147	T7-*yrkP*-6his; amp^R	*yrkP* amplified with LG302/LG303 and digested with Nde1/Xho1 and ligated into pETDUET	"
pLG148	T7-*yvcP*-6his; amp^R	*yvcP* amplified with LG304/LG305 and digested with Nco1/Xho1 and ligated into pETDUET	"
pLG149	T7-*cssR*-6his; amp^R	*cssR* amplified with LG306/LG307 and digested with Nde1/Xho1 and ligated into pETDUET	"
pLG158	T7-*phoP*-6his; amp^R	*phoP* amplified with LG318/LG319 and digested with Nde1/Xho1 and ligated into pETDUET	"
pLG178	T7-*yvrH*-6his; amp^R	*yvrH* amplified with LG338/LG339 and digested with Nco1/Xho1 and ligated into pETDUET	"
pLG199	T7-*lytT*-6his; amp^R	*lytT* amplified with LG321/LG322 and digested with Nhe1/BamH1 and ligated into pET11a	"
pSFP37	T7-6his-*prkC*; amp^R	Cytoplasmic domain of *prkC* including juxtamembrane region amplified using SFP59/SFP60 and digested with Nco1/Pst1 and ligated into pETDUET	

For SOE products, outer primers used to SOE fragments together are indicated in **bold.**

kan, kanamycin; spec, spectinomycin; amp, ampicillin; cm, chloramphenicol

References

66. Guerout-Fleury AM, Shazand K, Frandsen N, Stragier P (1995) Antibiotic-resistance cassettes for Bacillus subtilis. Gene 167: 335-336.
67. Guerout-Fleury AM, Frandsen N, Stragier P (1996) Plasmids for ectopic integration in Bacillus subtilis. Gene 180: 57-61.
68. Middleton R, Hofmeister A (2004) New shuttle vectors for ectopic insertion of genes into Bacillus subtilis. Plasmid 51: 238-245.
69. Ben-Yehuda S, Rudner DZ, Losick R (2003) RacA, a bacterial protein that anchors chromosomes to the cell poles. Science 299: 532-536.
70. D'Elia MA, Millar KE, Bhavsar AP, Tomljenovic AM, Hutter B, et al. (2009) Probing teichoic acid genetics with bioactive molecules reveals new interactions among diverse processes in bacterial cell wall biogenesis. Chem Biol 16: 548-556.
