# Supplementary material for: The Eukaryotic-Like Ser/Thr Kinase PrkC Regulates the Essential WalRK Two-Component System in Bacillus subtilis
Source: PLoS Genet. 2015 Jun 23;11(6):e1005275. doi: 10.1371/journal.pgen.1005275 (PMC4478028; doi:10.1371/journal.pgen.1005275)
Supplement: S4 Table — (PDF) [file pgen.1005275.s010.pdf]

S4 Table: Oligos used in this study

Name	Sequence (5'-3')
PywaC-luxABCDE-u1	TTAAAAACTGGTCTGATCGGATCCTAGAAGCTTGCCTCATACG GGATTG
PywaC-luxABCDE-l1	CCGATGATAAGCTGTCAAACATGAGAATTCAACTATCAAACGC TTCG
BamHI-PyjeA-u1	GGCAGGATCCGGACGCGGATAATAATAAGGCTG
SalI-PyjeA-l1	GGCAGTCGACCGTAGCCATTTCGATTTACTC
BamHI-PyoeB-u1	GGCAGGATCCCAAGAGGTTCTTGTGATGTTTCC
SalI-PyoeB-l1	GGCAGTCGACCAAAATCTCTTTCTCTTATGTTAC
BamHI-PyocH-u1	GGCAGGATCCGAAATACAGGCTTATGCAAGGATG
SalI-PyocH-l1	GGCAGTCGACGTTAGCTTTTTATTGGCGGCTAACAGG
LG66	GGCCGGATCCGAGAACCGGCCCTGCGGC
LG67	GGCCGAATTCTTA**TTTGTCGTCGTCGTCTTTATAGTC**GTCCT GTTCTGGGTTTCTCAA (bold is FLAG+STOP)
LG129	GGCCCATATGGAGCTTGCGAAAGGGAATTTC
LG130	GCTGCATATGGATAAAAAGATCCTTGTAG
LG134	GGCCGACGTCTTAGTGATGGTGATGGTGATGCGCTTCATCC CAATCATCC
LG135	CCAGCTCGAGTTAGTGATGGTGATGGTGATGGTCCTGTTCT GGGTTTCTCAA
LG174	GCTGGAATTCCATTTTGATTTTCCTCCTGC
LG177	GTCGGGTACCCACGCGAAGCACCGTTAATT
LG178	TGCGGGATCCCGCATATAAGTTGGTCTGAG
LG179	GTCGGTCGACATGAATAAGGTTGGTTTTTTTCGG
LG189	GCTGATGACTATGTCGCAAAACCATTCAGC
LG190	GCTGAATGGTTTTGCGACATAGTCATCAGC
LG225	GTGCTGAATGGTTTTGAGACATAGTCATCAGCAC
LG242	CTGATGACTATGTCTCAAAACCATTCAG
LG298	GCTGCATATGAAAATATTAATGATAGAAG
LG299	ATGTCTCGAGTTAGTGATGGTGATGGTGATGATCTTCATCAA ATTTATACC
LG300	ACTACCATGGCGGAAAAAGGACACATATTAAT
LG301	ATGTCTCGAGTTAGTGATGGTGATGGTGATGCCCCTTAATG GCATAGCC
LG302	GCTGCATATGGCCTATCGAATATTAGTCGT
LG303	ATGTCTCGAGTTAGTGATGGTGATGGTGATGAGATGCGCCA AATCGATA

LG304	ACTTCCATGGCGTATCGGATTTTGCTTGTGGAAGATG
LG305	ATGTCTCGAGTTAGTGATGGTGATGGTGATGACATTCCGCT TCATCCTTC
LG306	GCTACATATGTCATACACCATTTATCTAGTTG
LG307	ATGTCTCGAGTTAGTGATGGTGATGGTGATGTGATGACATC ATCCTGTAG
LG318	CCAGCTCGAGTTAGTCCTGTTCTGGGTTTCTCAA
LG319	ATGTCATATGAACAAGAAAATTTTAGTTG
LG321	TCATGGCTAGCCTCAGGGTGTTAATAGTTGATG
LG322	ACGCGGATCCTTAGTGATGGTGATGGTGATGAATATGGAGC AGCTTTTTC
LG318	CCAGCTCGAGTTAGTCCTGTTCTGGGTTTCTCAA
LG319	ATGTCATATGAACAAGAAAATTTTAGTTG
LG321	TCATGGCTAGCCTCAGGGTGTTAATAGTTGATG
LG322	ACGCGGATCCTTAGTGATGGTGATGGTGATGAATATGGAGC AGCTTTTTC
LG338	ACTACCATGGAAAATGCGTCAATTTTAATCG
LG339	AAAACTGCAGGAATAAGGTTGGTTTTTTTCGGTC
LG420	GCTCTAGACGACCAAAATCAAGCACGTT
LG421	CGCGGATCCTATTGTCATGCTGGTCAACT
LG422	ACGCGTCGACATAAACAACTGGCGATAAAG
LG423	CGGGGTACCAAGCGGGTGGGTAATGGTTC
SFP59	TATCCATGGCTCATCATCATCATCATCATGCTCTAATCGGCA AGCGGATCAGC
SFP60	TATCTGCAGTTATCATTTTCTTTTGCCGTTCTTC

Note: underlined sequences denote restriction sites.
