# Supplementary material for: The Eukaryotic-Like Ser/Thr Kinase PrkC Regulates the Essential WalRK Two-Component System in Bacillus subtilis
Source: PLoS Genet. 2015 Jun 23;11(6):e1005275. doi: 10.1371/journal.pgen.1005275 (PMC4478028; doi:10.1371/journal.pgen.1005275)
Supplement: S1 Text — (PDF) [file pgen.1005275.s011.pdf]

S1 Text: Supplementary Materials and Methods

Expression and Purification of 6xHis-tagged recombinant proteins

Recombinant proteins were expressed in *E. coli* from strains listed in Table S2. Details of plasmid construction are listed in Table S3. For expression, cultures were grown overnight at 37ºC in LB supplemented with ampicillin (100 µg/ml). The following morning they were diluted 1:500 into fresh media and grown at 37°C to an OD_{600} of 0.7-1. Cultures were then induced with 1mM IPTG for 3 hours at room temperature with aeration. Cultures were centrifuged at 5,000 x g for 10 min to pellet cells and stored at -80°C prior to purification. Pre and post-induction lysates were run on an SDS-PAGE gel to confirm protein expression.

Frozen pellets were thawed and resuspended in lysis buffer (50 mM Tris pH 7.5, 300 mM NaCl, 30 mM imidazole, 1 mg/ml lysozyme, and 1 mM PMSF) followed by incubation on ice with intermittent vortexing for at least 30 min. Lysates were then sonicated with 9 cycles of 5 sec followed by 30 sec of rest. After sonication, 5µg/ml of DNase1 and 3mM $MgCl_2$ (final concentrations) were added to the lysates, followed by gentle mixing for 15 minutes at 4°C. Lysates were cleared by centrifugation at 15,000 x g for 30 min at 4°C. Ni-NTA resin (Qiagen) was equilibrated with wash buffer (50 mM Tris pH 7.5, 300 mM NaCl, and 30 mM imidazole). Cleared lysates were incubated with Ni-NTA resin in empty protein columns (Pierce) at 4°C for at least 1 hour. Lysates were then allowed to flow through the column by gravity, followed by three washes with 10 column volumes of wash buffer. Proteins were eluted using increasing concentrations of imidazole in elution buffer (50 mM Tris pH 7.5, 300 mM NaCl, and 100-500 mM imidazole). Proteins were loaded onto SDS-PAGE and elution fractions chosen to dialyze overnight. Proteins were dialyzed overnight using 10 kDa MWCO mini-dialysis cups (Pierce) at 4°C in 50 mM Tris pH 7.5, 150 mM NaCl, 5% glycerol, and 1 mM DTT.

Analysis of *in vitro* WalR phosphorylation

In-gel Digestion of Coomassie-stained Bands: Gel bands were transferred to pre-digested tubes and 100µl of a 0.01M DTT and 0.1M Tris pH 8.5 solution was added. The tube was placed in a heating block at 55° for 1-2h. After cooling the tube to RT, the liquid was removed and replaced with 10 µl of a solution composed of 0.03M iodoacetamide and 0.1M Tris, pH 8.5. Incubation was for 30 min in the dark after which the liquid was removed and the gel was washed as described below. Gel bands were prepared for digestion by washing twice with 200 µl 0.05M Tris, pH 8.5 and 30% acetonitrile for 20 min with shaking, and once with 100µl acetonitrile for several minutes until the gel was opaque white. After removing the acetonitrile, the gel pieces were dried for 20-30 min in a Speed-Vac concentrator. Gels were digested by adding 0.1 mg modified trypsin (sequencing grade, Roche Molecular Biochemicals, Indianapolis, IN) in 50 µl 0.025M Tris, pH 8.5. The tubes were placed in a heating block at 32° and left overnight. Peptides were extracted with 2x 50 µl 50% acetonitrile/2% TFA and the combined extracts were dried in a Speed-Vac and dissolved in 20 µl 0.1% formic acid. 5 µl aliquots were analyzed by LC-MS/MS.

ESI-LC-MS/MS: LC-MS/MS analysis was done on a Waters Ultima Q-Tof hybrid quadrupole/time-of-flight mass spectrometer with a nanoelectrospray source. Capillary voltage was set at 1.8kV and cone voltage 32V; collision energy was set according to mass and charge of the ion, from 18eV to 50eV. Chromatography was performed on an LC Packings HPLC with a Dionex C18 PepMap column (15 cm x 75 mm) using a 90-minute linear acetonitrile gradient with flow rate of 200 nl/ min. Raw data files were processed using the MassLynx ProteinLynx software and .pkl files were submitted for searching at www.matrixscience.com using the Mascot algorithm. Search parameters were as follows: Database, SwissProt 2010_10; taxonomy, Bacillus subtilis; enzyme, trypsin; variable modifications, Carbamidomethyl (C),

Oxidation (M), Phospho (ST), Phospho (Y); max missed cleavages, 2; peptide mass tolerance, 1.2 Da; fragment mass tolerance, 0.6 Da.

Analysis of *in vivo* WalR phosphorylation

In-Gel Protein Digestion: Purified proteins were separated using precast 4–20% Tris-Glycine SDS-PAGE gels (1.0 mm thick) (Life Technologies, Carlsbad, CA). The protein gels were stained with Coomassie Blue and gel bands containing WalR were cut, destained, reduced, alkylated, and dried for in-gel digestion. The dried gel pieces were rehydrated and digested in 80 µL of 12.5 ng/µL Trypsin Gold/50 mM ammonium bicarbonate at 37 °C overnight. After the digestion was complete, condensed evaporated water was collected from tube walls by 5 s centrifugation using benchtop microcentrifuge (Eppendorf, Hauppauge, NY). The gel pieces and digestion reaction were mixed with 50 µL 2.5% TFA and rigorously mixed for 15 min. The solution with extracted peptides was transferred into a fresh tube. The remaining peptides were extracted with 80 µL 70% ACN/5% TFA mixture using rigorous mixing for 15 min. The extracts were pooled and dried to completion (1.5–2 h) in SpeedVac. The dried peptides were reconstituted in 30 µL 0.1% TFA by mixing for 5 min and stored in ice or at −20°C prior to analysis.

LC-MS/MS Analysis: Samples were centrifuged at 14000–16000 × *g* for 10 min to remove particulate material. Five microliters of each sample were injected into a self-packed fused silica nano column, which had been pulled to a 5 µm i.d. tip using a P-2000 CO_2 laser puller (Sutter Instruments), then packed with 5 cm of 3 µm C18 reverse phase (RP) particles (Magic *C18* AQ 3 µm (Michrom; Bioresources, Auburn, CA,) and equilibrated in 5% acetonitrile, 0.1 % formic acid (Buffer A). The LC system was an Eksigent nano2D LC run at 0.5µl/min. Mobile phase A was 5% acetonitrile, 0.1 % formic acid (Buffer A), mobile phase B was 98% acetonitrile/0.1%

formic acid (Buffer B). A linear gradient from 2% mobile phase B to 40% mobile phase B over 90 minutes was used. Then, we ramped up 80% mobile phase B for 3 minutes and maintained the gradient at 80% mobile phase B for additional 3 minutes. The application of a 2.5 kV distal voltage electrosprayed the eluting peptides directly into a LTQ-Orbitrap XL mass spectrometer equipped with a nano-LC electrospray ionization source (ThermoFinnigan). Full MS spectra were recorded on the peptides over a 400 to 2000 m/z range by the Orbitrap, followed by either MS/MS generated from the targeted precursor mass (*in vivo* detection) or five tandem mass (MS/MS) events sequentially generated by LTQ in a data-dependent manner on the first, second, third, and fourth most intense ions selected from the full MS spectrum (at 35% collision energy) (synthetic peptide). Mass spectrometer scan functions and HPLC solvent gradients were controlled by the Xcalibur data system (ThermoFinnigan, San Jose, CA).

Database search and interpretation of MS/MS datasets: Tandem mass spectra were searched against a concatenated target-decoy database containing the forward and reverse sequences of the target database for false discovery rate calculation (FDR) using the data search algorithm specialized for post-translational modification search, InspecT. The reference proteome set of Bacillus subtilis (strain 168) was downloaded from UniProt website (http://www.uniprot.org/). The forward database contains the reference proteome set and 124 common contaminant proteins. The search algorithm was configured to specify the following parameters: precursor tolerance, 10ppm; fragment tolerance, 0.5 Da; static modification, cysteine carbamidomethylation, fully tryptic status, and 1% FDR. For phosphorylation, 79.9663 STY was chosen as the differential modification search.
